# Parameter estimation of hyper-spherical diffusion models with a time-dependent threshold: An integral equation method

**DOI:** 10.3758/s13428-025-02810-3

**Published:** 2025-09-10

**Authors:** Amir Hosein Hadian Rasanan, Nathan J. Evans, Jamal Amani Rad, Jörg Rieskamp

**Affiliations:** 1 Department of Psychology, University of Basel, Basel, Switzerland; 2 Department of Psychology, University of Liverpool, Liverpool, UK; 3 School of Psychology, University of Queensland, Queensland, Australia; 4 Choice Modelling Centre and Institute for Transport Studies, University of Leeds, Leeds, UK

**Keywords:** Decision making, Continuous option space, Parameter estimation, Collapsing thresholds, Sequential sampling models

## Abstract

Over the past several decades, decision-making research has been dominated by the study of binary choice tasks, with key models assuming that people remain equally cautious regardless of how long they have spent on the choice problem. Recent research has begun to place a greater focus on studying tasks with a continuous-response scale, as well as models that allow for decreases in caution over decision time; however, these research topics have remained separate from one another. For instance, proposed models of continuous-response scales have assumed constant caution over time, and studies investigating whether caution decreases over time have focused on binary choice tasks. One reason for this separation is the lack of methodology for estimating the parameters of the decision models with time-dependent parameters for continuous responses. This paper aims to provide a stable and efficient parameter estimation technique for hyper-spherical diffusion models with a time-dependent threshold. Here, we propose an integral equation method for estimating the first-passage time distribution of hyper-spherical diffusion models. We assessed the robustness of our method through parameter recovery studies for constant and time-dependent threshold models, with our results demonstrating efficient and precise estimates for the parameters in both situations.

## Introduction

People often make decisions between continuous alternatives (Hadian Rasanan et al., 2024). These continuous-response decisions can occur in many everyday situations. For instance, when making investment decisions, people decide how much of their financial resources they want to invest in a particular fund or stock (Olschewski et al., 2021; Rieskamp et al., 2003). Alternatively, people sometimes decide about the price they are willing to sell an item (such as a car) (Izakson et al., 2024; Kvam & Busemeyer, 2020). In experimental settings, continuous-response decision tasks are often simplification of everyday situations, such as visual working memory studies where people are required to recall the direction, orientation, location, or color of a stimulus on a continuous scale (e.g., Blake et al., 1997; Fennell & Ratcliff, 2023; Prinzmetal et al., 1998; Schurgin et al., 2020; Smith, 2016; Wilken & Ma, 2004; Zhang & Luck, 2008). Importantly, continuous-response decisions provide much greater information than their discrete decision counterparts (Sokratous et al., 2023), which has resulted in their increasing popularity within experimental psychology. For instance, the continuous-response has already been used frequently in different studies on numerical cognition (Cohen et al., 2011; Ratcliff & McKoon, 2020, 2024; Siegler & Opfer, 2003; Slusser et al., 2013), motion perception (Bae & Luck, 2019; Barbieri et al., 2023; Chetverikov & Jehee, 2023; Green & Pratte, 2022; Manning et al., 2022; Mc Keown et al., 2023; Smith et al., 2023a), and orientation perception (Kvam, 2019). Since continuous-response decisions can also be applied to many real-life situations and provide a lot of information about cognitive processes, they represent an important empirical research topic (Hadian Rasanan et al., 2024).

Within decision-making research, sequential sampling models (SSM; Laming, 1968; Ratcliff, 1978; Stone, 1960) have become a dominant theoretical framework for understanding how people make decisions in situations with two or several alternatives (Busemeyer et al., 2019; Forstmann et al., 2016; Heathcote & Matzke, 2022; Ratcliff et al., 2016). These models assume that the decision-maker samples noisy evidence from the environment at some rate (often referred to as the drift rate), and once the amount of integrated evidence for one option exceeds a threshold (known as the decision threshold), a decision is made. Recently, SSMs have been extended for continuous-response decisions (e.g., Kvam et al., 2023; Ratcliff, 2018; Smith, 2016). Various proposals for this purpose have been made, such as the 2-dimensional circular diffusion model (Smith, 2016) and its *n*-dimensional generalization, the hyper-spherical diffusion model (HSDM; Smith & Corbett, 2019), the spatial continuous diffusion model (SCDM; Ratcliff, 2018; Ratcliff & McKoon, 2020), the radial basis leaky competing accumulator model (Hadian Rasanan et al., 2022), and the multiple anchor accumulation model (Kvam et al., 2023). While each variant has different strengths and weaknesses (see Kvam and Turner (2021) and Hadian Rasanan et al. (2024) for a detailed comparison between the models), the current manuscript focuses on the HSDM for several reasons. First, the mathematical tractability of the likelihood function in the HSDM makes it easy to handle and to estimate. Second, the HSDM provides a direct mapping between the response scale in the model (i.e., a continuous decision threshold) and the experimental response scale (i.e., a circle or sphere of response options), such as color wheels typically used in visual working memory tasks (Smith, 2016; Zhang & Luck, 2008). Finally, the HSDM had been successful in describing various experimental findings, such as working memory (Smith, 2016; Souza & Frischkorn, 2023), source memory (Osth et al., 2023; Zhou et al., 2021, 2023), random dot motion (Smith et al., 2023a), orientation judgment (Kvam, 2019), and even pricing tasks (i.e., pricing as seller or buyer Izakson et al., 2024; Kvam & Busemeyer, 2020; Sokratous et al., 2023). Thus, in the current work, we will focus on the HSDM and propose a dynamic decision threshold contrary to previous implementations of all continuous-response decision SSMs that were restricted to constant decision thresholds.

Traditionally, SSMs have assumed that people’s decisions can be best described by assuming fixed decision thresholds. Accordingly, it is assumed that people always accumulate the same amount of evidence, regardless of the time required, to make a decision. Recently, it has been argued that the decision threshold decreases over time, so that less evidence is required after some time has elapsed (Diederich & Busemeyer, 2006; Drugowitsch et al., 2012). Specifically, these collapsing thresholds – and similar proposals, such as urgency signals (Cisek et al., 2009; Trueblood et al., 2021) – suggest that after some time, people feel urged to make a decision. Collapsing thresholds have also been motivated by normative considerations; specifically, when the quality of incoming evidence is unknown and/or variable between options, then collapsing thresholds provide the most efficient information processing strategy (i.e., the strategy that maximizes the reward rate) (Drugowitsch et al., 2012; Fudenberg et al., 2018; Frazier & Yu, 2007; Malhotra et al., 2018; Tajima et al., 2016). However, the empirical evidence for collapsing thresholds – or other decision strategies where urgency increases over time – has been mixed. Specifically, whereas some studies have found evidence in favor of collapsing thresholds (Bhui, 2019; Olschewski et al., 2025; Palestro et al., 2018), others found the opposite (Milosavljevic et al., 2010; Smith & Ratcliff, 2022; Voskuilen et al., 2016), or found that people only seem to adopt collapsing thresholds under certain task conditions, such as when there are decision deadlines or an emphasis on urgency (Evans & Hawkins, 2019; Evans et al., 2020; Hawkins et al., 2015). In general, whether people feel more urged over time to make a decision continues to be debated in decision-making research.

Previous research on collapsing thresholds for SSMs has focused on two-alternative forced choice situations. However, decreasing thresholds could also be beneficial for continuous-response alternatives. For example, empirical studies on continuous-response decisions have shown that decisions became more variable under time pressure, which is consistent with the idea of collapsing thresholds (Kvam, 2019). One primary problem for assuming collapsing thresholds for SSMs applied to continuous-response decisions is the lack of a *methodology* for estimating the models. Specifically, many SSMs that have been proposed for continuous-response decisions are already highly complex, with some not having an exact/analytical likelihood function, requiring time-consuming simulation-based methods for estimation. Contrary to other SSMs, the HSDM has an analytical solution; thus, adding a time-varying threshold appears more attractive than for models that require simulation.

In this work, we develop and demonstrate an integral equation method for approximating the first-passage time distribution of the HSDM, which enables efficient estimation of the model’s likelihood both with and without time-dependent thresholds. Specifically, in the integral equation method, the first-passage time distribution of the one-dimensional Wiener process is obtained by approximating the solution of a Volterra integral equation using a numerical scheme (Smith, 2000). Durbin (1971) was the first to employ this method to study the boundary-crossing behavior of the Wiener process with time-dependent absorbing boundaries. Since then, the method and some extensions have been utilized to study the first-passage time properties of various stochastic processes (e.g., Buonocore et al., 1987, 1990; di Crescenzo et al., 2024; Giorno & Nobile, 2021), including the estimation of the parameters of the diffusion model (Evans et al., 2020; Heath, 1992; Jones & Dzhafarov, 2014; Smith, 1995, 2023; Smith & Ratcliff, 2009, 2022; Smith & Lilburn, 2020; Smith et al., 2014; Voskuilen et al., 2016; Zhang et al., 2014). The main advantage of the integral equation approach over the other estimation methods is the simplicity and efficiency of the method, which we will also discuss further in the discussion section.

The aim of this paper is to propose an integral equation method for approximating the first-passage time distribution of HSDM and estimating the parameters of the model based on this method. However, we do not aim to provide empirical evidence for the model, although we believe it offers a plausible way of describing human decision-making. The remainder of this paper is organized as follows. The mathematical properties of HSDM are presented in Section “Hyper-spherical diffusion model”. Furthermore, this section highlights the problem with the analytical form of the first-passage time distribution based on Bessel functions. Section “Integral equation method for HSDM” presents the integral equation method for approximating the first-passage time distribution of HSDM. Sections “Simulation study: Method” and “Simulation study: Results” present a simulation study to assess the parameter recovery of the HSDM using the proposed integral equation method, with Section “Simulation study: Method” providing the simulation methodology and Section “Simulation study: Results” providing the simulation results. Finally, Section “General discussion” provides a general discussion about the utility of the method in empirical studies and potential future directions.

## Hyper-spherical diffusion model

**Fig. 1:**
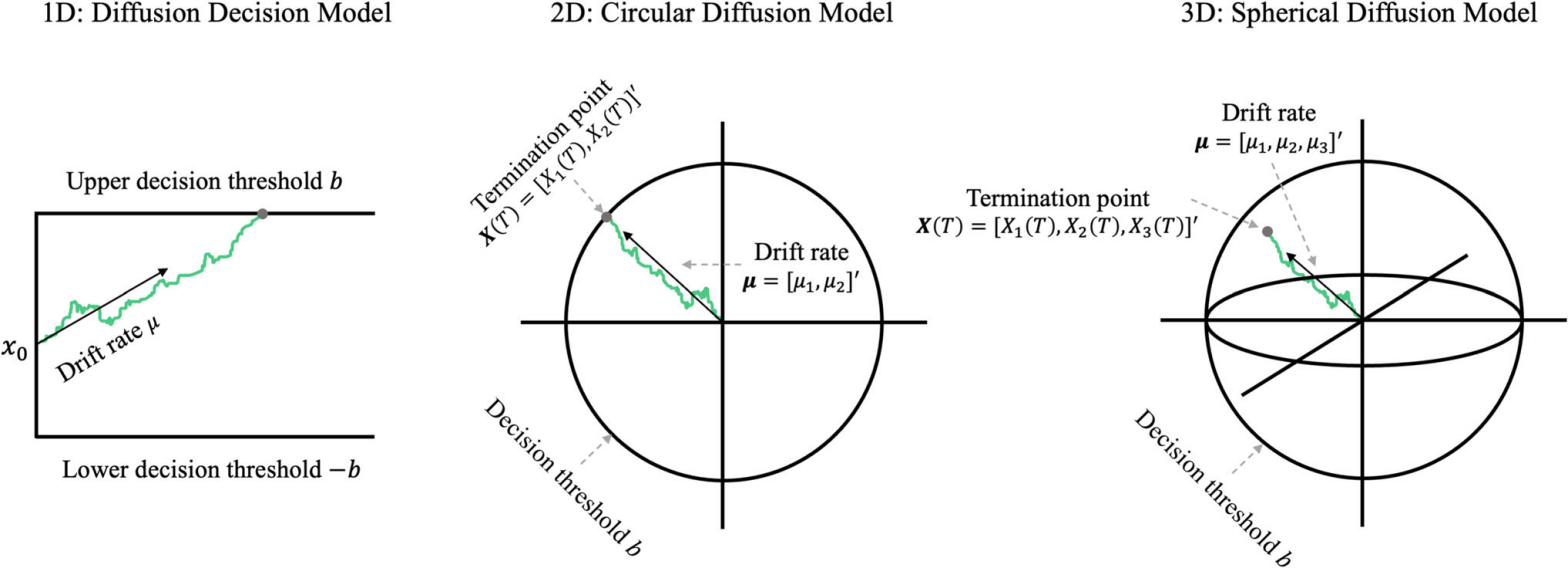
The schematic plots of a one-dimensional (*left panel*; diffusion decision model), a two-dimensional (*middle panel*; circular diffusion model), and a three-dimensional (*right panel*; spherical diffusion model) diffusion model. The figure is adapted from Smith and Corbett (2019)

HSDM is the *n*-dimensional extension of the well-known diffusion decision model (DDM; Ratcliff, 1978; Ratcliff & McKoon, 2008). The DDM is designed to explain the decision process in two-alternative choice scenarios and has been applied to a wide range of fields that involve decision making (for a review on the DDM and further applications of the model, see Evans & Wagenmakers, 2020; Forstmann et al., 2016; Ratcliff et al., 2016). Specifically, the DDM assumes that a single relative accumulator (*X*(*t*)) integrates noisy evidence within a one-dimensional evidence space with a constant mean rate called drift rate ($\mu$) until the accumulator meets one of the lower or upper thresholds (i.e., $|X(t)|\ge b$), and then a corresponding decision is made. In this model, the drift rate’s magnitude indicates how strong the input signal is (i.e., signal-to-noise ratio). In other words, a stronger input signal yields a higher drift rate value, which predicts faster and more accurate responses. The threshold value indicates how much information is required to make a decision. A higher threshold value requires more evidence to be accumulated for making a decision, which predicts slower and more accurate responses. The DDM also contains a parameter for the starting point of the accumulation (i.e., the location of the accumulator at the beginning, $X(0)=x_0$), which reflects any a priori bias that the decision maker has towards either option. The accumulation process in this model can be formulated by a one-dimensional Wiener process (also known as Brownian motion) as follows:

1 $$\begin{aligned} dX(t) = \mu dt + \sigma dW(t), ~~~~~~~~ X(0)=x_0, \end{aligned}$$

in which $\sigma$ is the diffusion coefficient and determines the level of noise during the evidence accumulation, and *dW*(*t*) is the Wiener process. Finally, the accumulation process considered in (1) only explains the decision time. However, the response time also reflects some additional components that are not directly related to the decision-making process, such as encoding time and motor execution time. Accordingly, the DDM considers total encoding and motor time as an additional parameter called non-decision time ($t_0$). Therefore, DDM can predict response time by summing up the decision time predicted by the accumulation process (1) and the non-decision time. The left panel of Fig. 1 illustrates how the proposed decision process in the DDM operates.

The HSDM (Smith, 2016; Smith & Corbett, 2019) extends the one-dimensional Wiener process in the DDM to a multi-dimensional Wiener process, where the accumulation process occurs within a hyper-sphere (e.g., a circle for a two-dimensional process, a sphere for a three-dimensional process). In other words, the evidence accumulation process in HSDM is represented by an *n*-dimensional vector-valued Wiener process as (2) fluctuating within an *n*-dimensional hyper-sphere:

2 $$\begin{aligned} \textbf{dX}(t) = \pmb {\mu } dt + \pmb {\sigma } \textbf{dW}(t), \end{aligned}$$

where the accumulator $\textbf{X}(t)$ is a vector with components $\textbf{X}(t) = \big [X_1(t), \dots , X_n(t)\big ]'$.^1^$\pmb {\mu } = \big [\mu _1, \dots , \mu _n\big ]'$ is the drift vector, and each $\mu _i$ shows the drift rate for the *i*-th axis (i.e., each direction of accumulation). $\pmb {\sigma } = \sigma I$ is the covariance matrix where *I* represents $n \times n$ identity matrix.^2^$\textbf{dW}(t) = \big [dW_1(t), \dots , dW_n(t)\big ]'$ denotes an *n*-dimensional (vector-valued) Wiener process. Thus, in a matrix format, the stochastic process (2) can be represented as follows:

3 $$\begin{aligned} \begin{bmatrix} dX_1(t)\\ \vdots \\ dX_n(t) \end{bmatrix} = \begin{bmatrix} \mu _1 \\ \vdots \\ \mu _n \end{bmatrix} dt + \begin{bmatrix} \sigma & & 0 \\ & \ddots & \\ 0 & & \sigma \end{bmatrix} \begin{bmatrix} dW_1(t) \\ \vdots \\ dW_n(t) \end{bmatrix}. \end{aligned}$$

The model assumes that the accumulation process starts from the origin (i.e., $\textbf{X}(0) = [0, \dots , 0]'$)^3^ and continues to accumulate until the Euclidean (i.e., linear) distance of the accumulator from the origin becomes greater than the decision threshold (i.e., $\sqrt{\sum _{i = 1}^{n}X^2_i(t)} \ge b$; accumulator crosses the circumference of the hyper-sphere). Therefore, when the process hits a point on the surface of the hyper-sphere (e.g., a point on the perimeter of the circle or sphere), the process will stop, and the corresponding point on the response scale will be selected. Therefore, the evidence space is $\mathbb {R}^n$, and the decision threshold is a hyper-sphere. For instance, the decision threshold for the two-dimensional model is a circle, and for the three-dimensional model is a sphere with a radius *b*. Figure 1 illustrates a two-dimensional (circular diffusion model; center panel) and a three-dimensional (spherical diffusion model; right panel) HSDM.

Similar to the DDM, the decision threshold (i.e., the radius of the hyper-sphere; *b*) represents how people could adjust their speed-accuracy tradeoff, by reducing the radius to become faster or increasing the radius to become more cautious. We should note that in continuous-response tasks, there is no binary definition for accuracy, and usually, it is measured by the absolute/square deviation from the correct response. Consequently, the “speed–accuracy" tradeoff can be thought of as a more generalized “speed–variance" tradeoff for continuous-response tasks, where a higher decision threshold results in slower and more consistent decisions, and a lower decision threshold results in faster responses with greater variability in responses. However, as with the DDM, response time and response consistency are also highly influenced by the drift rate, which in the case of the HSDM is the length of the drift rate (i.e., $\Vert \pmb {\mu }\Vert = \sqrt{\sum _{i=1}^{n} \mu _i^2}$). A higher value for the drift length means a stronger input signal and results in faster and more accurate responses. However, a smaller value for the drift length means a weaker input signal, and therefore, predicts slower and more variable responses. To measure the precision of the decision maker, HSDM introduces $\kappa$, which is (Smith, 2016):

$$\kappa = \frac{b \times \Vert \pmb {\mu }\Vert }{ \sigma ^2}.$$

This relation indicates that the decision maker’s precision is the product of the strength of the input signal (i.e., stimulus quality) and the amount of evidence required for making a decision.

**Fig. 2:**
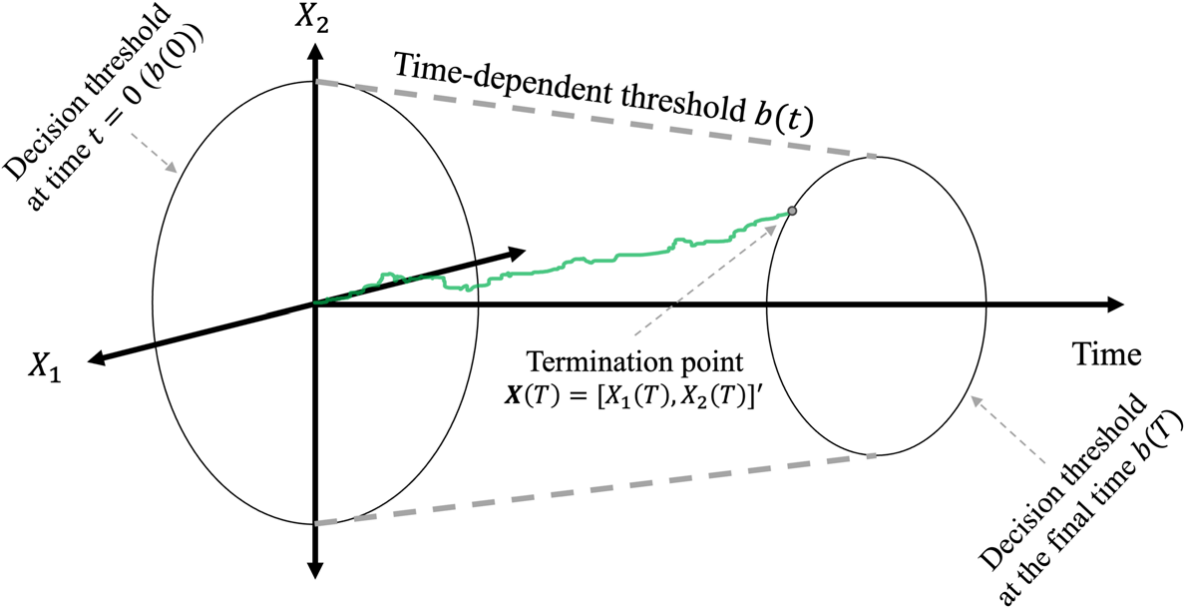
A schematic view of two-dimensional HSDM (circular diffusion model) with a linear collapsing threshold. The radius of the circular decision threshold shrinks linearly over time

The original HSDM (Smith, 2016; Smith & Corbett, 2019) was proposed based on the assumption that the decision threshold remains constant over time. However, since the accumulation process is costly (e.g., Cisek et al., 2009; Drugowitsch et al., 2012; Frazier & Yu, 2007), time-dependent threshold HSDM can be a plausible extension of the original HSDM. In contrast to the constant-threshold models, a time-dependent threshold (i.e., *b*(*t*)) means that the amount of evidence required for making a decision changes over time. One common dynamic for the decision threshold, as discussed in the introduction, is the collapsing dynamics, which reflects the idea of increasing urgency over time. In the HSDM, a collapsing threshold operates by decreasing the radius of the hyper-sphere over time, which implies that later decisions are more random (i.e., decisions with longer response times are also more variable). Figure 2 illustrates an example of the circular diffusion model with a linear collapsing threshold.

### Relation to response time and choice

To estimate the HSDM model with a constant threshold on empirical data and find the optimal model’s parameters, Smith (2016) has utilized the Girsanov change-of-measure theorem (Girsanov, 1960). This theorem provides a relation between the probability distributions of zero-drift and nonzero-drift processes.^4^ Therefore, Smith (2016) first obtained the first-passage time distribution for the zero-drift process and then, by using the Girsanov change-of-measure formula, obtained the joint distribution of response time and choice for the nonzero-drift process.

It is well known that the Euclidean distance from the origin of a zero-drift *n*-dimensional Wiener process (i.e., $R(t) = \sqrt{X^2_1(t) + \cdots + X^2_n(t)}$) is a Bessel process which satisfies the following stochastic differential equation:

4 $$\begin{aligned} dR(t) = \frac{n-1}{2R(t)} dt + \sum _{i=1}^{n}\frac{W_i(t)}{R(t)}dW_i(t), ~~~ n\ge 2. \end{aligned}$$

The first-passage time $T = \inf _{t>0}\{t: R(t) \ge b | R(0) = r_0 < b\}$ for this process has the following cumulative density function (Hamana & Matsumoto, 2013):

5 $$\begin{aligned} G(T) = 1 - \frac{1}{2^{\nu - 1} \Gamma (\nu + 1)} \sum _{k=1}^{\infty } \frac{j^{\nu -1}_{\nu , k}}{J_{\nu + 1}(j_{\nu , k})}\exp \Big \{-\frac{j^2_{\nu , k} \sigma ^2}{2b^2}T\Big \}, \end{aligned}$$

where $\nu = \frac{n-2}{2}$ and $J_{\nu }(z)$ is the Bessel function of the first kind of the order $\nu$ and is defined as follows (Bell, 2004):

$$J_{\nu }(z) = \sum _{k=0}^{\infty } \frac{(-1)^k(z/2)^{2k+\nu }}{k! \Gamma (k + \nu + 1)},$$

in which $\Gamma (.)$ is the well-known Gamma function defined as $\Gamma (s) = \int _{0}^{\infty } t^{s-1} e^{-t}dt$ for $s>0$. Also, $j_{\nu , k}$ is the *k*-th root of the Bessel function of the first kind of the order $\nu$ (i.e., $J_{\nu }(z)$).

**Fig. 3:**
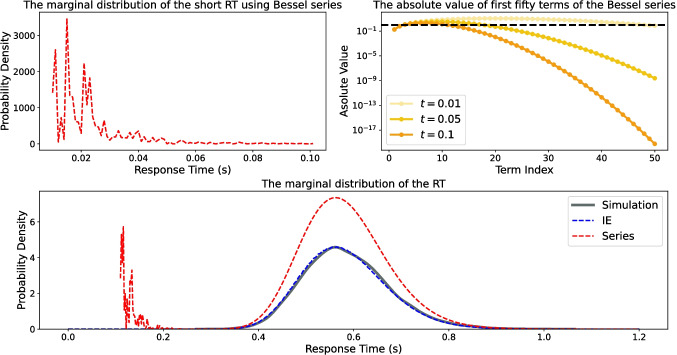
The marginal response time distribution for a circular diffusion model with drift rate $\pmb {\mu } = [6, 6]'$, diffusion coefficient $\sigma =1$, and decision threshold $b=5$. *Upper-Left panel*: The marginal distributions for short response times based on Bessel series estimation with 500 terms. *Upper-tight panel*: The absolute value of the first 50 terms of the Bessel series (6) for different time points. Each *dot* shows the absolute value of a single term. *Lower panel*: The marginal response time distribution of 10,000 simulated trajectories against the Bessel series and the integral equation estimations. The *red dashed line* represents the Bessel series estimation with 500 terms for the marginal distribution of response time longer than 0.1, and the *blue dashed line* shows the integral equation estimation

The cumulative density function, *G*(*T*), shows the probability that the process hits the fixed threshold *b* before time *T*. By definition, we know that the first-passage time distribution is the temporal differentiation of the cumulative first-passage time distribution (5). So, the first-passage time density of the zero-drift process is defined as follows (Hamana & Matsumoto, 2013):

6 $$\begin{aligned} g(T) = \frac{\sigma ^2}{2^{\nu } b^2 \Gamma (\nu + 1)} \sum _{k=1}^{\infty } \frac{j^{\nu +1}_{\nu , k}}{J_{\nu + 1}(j_{\nu , k})}\exp \Big \{-\frac{j^2_{\nu , k} \sigma ^2}{2b^2}T\Big \}. \end{aligned}$$

By using the Girsanov change-of-measure formula (see Appendix A), the joint distribution of response time and choice for the nonzero-drift process is obtained as follows (Smith, 2016; Smith & Corbett, 2019):

7 $$\begin{aligned} P(T, \varphi _1, ~\dots ~, \varphi _{n-1} |b , \pmb {\mu })= &  \exp \Big [\frac{1}{\sigma ^2}\big (\pmb {\mu }\cdot \textbf{X}(T)\big ) \nonumber \\ &  - \frac{1}{2\sigma ^2}\Vert \pmb {\mu }\Vert ^2 T\Big ]g(T), \end{aligned}$$

where *T* is the decision time, which is equal to the difference between response time and non-decision time (i.e., $T = RT - t_0$). Also, $\varphi _i$ ($i=1, ~\dots ~, n-1$) is the response angle with respect to *i*-th axis. For computing $\pmb {\mu }\cdot \textbf{X}(T)$, one can use the following Cartesian representation of choice:

- $$X_1(T) = b \cos (\varphi _1),$$
- $$X_2(T) = b \sin (\varphi _1) \cos (\varphi _2)$$, $$~~~~~~\vdots $$
- $$X_{n-1}(T) = b \sin (\varphi _1) \sin (\varphi _2)~ \dots ~\sin (\varphi _{n-2})\cos (\varphi _{n-1}),$$
- $$X_{n}(T) = b \sin (\varphi _1) \sin (\varphi _2)~ \dots ~\sin (\varphi _{n-2})\sin (\varphi _{n-1}).$$

The first-passage time distribution proposed in (6) only works for HSDMs with constant thresholds, and unfortunately, there is no analytical formulation for HSDMs with time-dependent thresholds. In addition, even for HSDMs with constant thresholds, this series has some computational issues and is unstable for some parameter values. Accordingly, even employing this series for HSDMs with a constant threshold can be problematic. The following section will elucidate the issues associated with this series. It is also worthwhile to mention that a simpler version of the circular diffusion model, called the EZ-circular diffusion model, has recently been proposed by Qarehdaghi and Amani Rad (2024), which has a closed-form solution for the parameters (i.e., does not need to compute the likelihood function). However, this model is limited to only two-dimensional cases with a constant threshold, and we cannot use this method for models with higher dimensions and/or time-dependent thresholds.

### The problems with the Bessel series

In some circumstances, estimating the first-passage time distribution based on the Bessel series (6) can be imprecise. This is particularly the case when the decision threshold or drift length is high (Smith et al., 2023b). The main problem with the proposed infinite series is that it generates some artificial spikes for short response times (Smith et al., 2023b) when the decision threshold or the drift length has high values. Basically, this estimation has two sources of error: a) series truncation and b) finite precision for calculating Bessel functions and Bessel roots. Figure 3 (upper-left panel) depicts an example of the imprecise estimation of the marginal distribution for short response times based on the Bessel series (6).

The first source of error is the series truncation. The Bessel series (6) is an infinite series, and we can only compute a finite number of terms for estimating the first-passage time distribution. Therefore, by computing a finite number of terms, a truncation error occurs in the estimation. This truncation error for short response times is larger than for long response times. Figure 3 (upper-right panel) shows the trend for the absolute value of the series terms, where the convergence is slower for shorter response times than for longer response times, and accordingly, the truncation error is larger. For instance, when $t=0.01$, most terms of the series are larger than one, and the convergence is too slow. This slow convergence rate for the short response times and the truncation error causes imprecise estimation of the first-passage time distribution for short response times. We should note that this issue also occurs for the standard DDM, and some researchers have tried to propose alternative series methods for short response times (e.g., Navarro & Fuss, 2009; Van Zandt, 2000; Van Zandt et al., 2000).

The second source of error is the finite floating-point precision in calculating Bessel functions and Bessel roots. As shown in (6), in order to calculate the first-passage time distribution, we need to calculate the value of Bessel functions and their roots. Unfortunately, there is no exact formulation for Bessel functions or their roots. Consequently, we can only calculate them with limited precision. In other words, each term in the series calculation contains a small error. Although these errors may be minimal, they can become significant when the series value is multiplied by the exponential term from the Girsanov change-of-measure theorem. See also Smith et al. (2023b) for a discussion on this issue.

Recently, Smith et al. (2023b) have considered this problem and proposed an asymptotic solution. The proposed method was based on estimating the first-passage time density for the short response time with an approximate explicit expression. Thus, by considering a splitting time point, the first-passage time distribution is estimated by an approximate explicit expression when the response time is shorter than the splitting time point and by the Bessel series when the response time is longer than the splitting time point. This method gives a stable estimation of the first-passage time distribution. However, finding the splitting point can be challenging, particularly since this point is not constant across different data sets and applications.

## Integral equation method for HSDM

To obtain the likelihood function for the HSDM with a time-dependent threshold, we have employed a similar approach to Smith (2016). First, we estimate the first-passage time distribution of zero-drift HSDM with a time-dependent threshold, and then, by utilizing the Girsanov theorem, we obtain the joint probability of response time and choice. To obtain the integral equation corresponding to the zero-drift *n*-dimensional Wiener process, instead of considering the distance from the origin, we consider the square of the distance from the origin $Y(t) = R^2(t) = X^2_1(t) + \cdots + X^2_n(t)$. This process is known as the squared-Bessel process. Then, by employing the Itô’s lemma for the derivative, we can derive the following one-dimensional stochastic differential equation for *Y*(*t*) (Göing-Jaeschke & Yor, 2003; Kersting et al., 2023; Revuz & Yor, 1999):

8 $$\begin{aligned} dY(t) = n dt + 2\sqrt{Y(t)}dW(t). \end{aligned}$$

This process is a special case of the Feller-type process (Feller, 1951) with a drift rate equal to *n*, infinite infinitesimal variance 4*Y*(*t*) and state-space $[0, +\infty )$.^5^ Since *Y*(*t*) is the square distance from the origin, then the first-passage time is defined by:

$$T = \inf _{t\ge 0}\{t: Y(t) \ge S(t)=b^2(t) | Y(0) = y_0 < S(0)\}.$$

*T* is the first time that $Y(T) \ge b^2(T)$. In other words, *T* is the first time the zero-drift *n*-dimensional Wiener crosses the time-dependent threshold *b*(*t*). Durbin (1971) showed that the first-passage time distribution of an Itô process satisfies the following Volterra integral equation of the first kind:

$$f[S(t), t| y_0, 0] \!=\! \int _0^t g[S(\tau ), \tau | y_0, 0] f[S(t), t| S(\tau ), \tau ] d\tau ,$$

where *f* stands for the free transition probability density function for the process *Y*(*t*) starting at $Y(0) = y_0$, defined as follows:

9 $$\begin{aligned} f [y, t|y_0, \tau ]= &  \frac{1}{2(t - \tau )}\big (\frac{y}{y_0}\big )^{(n-2)/4}\nonumber \\ &  \exp \big (-\frac{y+y_0}{2(t - \tau )}\big )I_{n/2 - 1}\big (\frac{\sqrt{y y_0}}{t-\tau }\big ). \end{aligned}$$

in which $I_{\nu }(z)$ is the modified Bessel function of the first kind of order $\nu$ and is defined as below (Bell, 2004):

$$I_{\nu }(z) = \sum _{k=0}^{\infty }\frac{(z/2)^{2k+\nu }}{k!\Gamma (k + \nu + 1)}.$$

While this integral equation is very simple, it has a singularity issue. In other words, as $t\rightarrow 0$, then the kernel function $f \rightarrow \infty$ and approximating the solution of this integral equation becomes intractable. Buonocore et al. (1987) has suggested a new integral equation form that resolves this singularity problem. The integral equation corresponding to a single threshold process is a Volterra integral equation of the second kind, formulated as follows (Buonocore et al., 1987):

10 $$\begin{aligned} g[S(t), t|y_0, 0]= &  - 2 \Psi [S(t), t, |y_0, 0] + 2\int _0^t g[S(\tau ),\nonumber \\ &  \tau |y_0, 0] \Psi [S(t), t | S(\tau ), \tau ] d\tau , \end{aligned}$$

in which $g[S(t), t|y_0, 0]$ is the first-passage time density of the process *Y*(*t*) crossing the time-dependent threshold *S*(*t*), and $\Psi$ is the kernel function which is defined based on the properties of the process. The kernel function for the Feller-type processes has already been obtained by Giorno et al. (1989). The authors showed that for a general form of stochastic process $dX(t) = A(x) dt + \sqrt{B(x)} dW(t)$ with the transition probability density function $f[x, t | y, \tau ]$, the kernel function $\Psi$ can be derived as follows (see lemma 1.1 and 1.2, Giorno et al., 1989):

11 $$\begin{aligned} \Psi [S(t), t | y , \tau ]&= \Bigg \{\frac{d}{dt}S(t) - A\big (S(t)\big ) + \frac{1}{2} \frac{d}{dt}B\big (S(t)\big ) \nonumber \\&\quad + \mathcal {K}(t)\Bigg \}f[S(t), t | y, \tau ]\\ \nonumber&\quad + \frac{1}{2} B\big (S(t)\big )\frac{\partial }{\partial x} f[x, t | y, \tau ] \Big |_{x = S(t)}, \end{aligned}$$

where $\mathcal {K}(t)$ is defined as follows:

12 $$\begin{aligned} \mathcal {K}(t) = \frac{1}{2} \Big (A\big (S(t)\big ) - \frac{d}{dt}S(t) - \frac{1}{4}\frac{d}{dx}B(x)\Big |_{x=S(t)} \Big ). \end{aligned}$$

In the case of stochastic process (8), $A(x) = n$, $B(x) = 4x$, and $f[x, t | y, \tau ]$ is defined in equation (9). Thus, by substituting these values for *A*(*x*), *B*(*x*), and $f[x, t | y, \tau ]$ into equations (11) and (12) and using the relation $\frac{d}{d z}I_{\nu }(z) = I_{\nu }(z) + \frac{\nu }{z} I_{\nu + 1}(z)$, the $\Psi$ function and $\mathcal {K}(t)$ for the process (8) are obtained as follows (see equation 6.5 in Giorno et al., 1989):

13 $$\begin{aligned} \Psi [S(t), t|y, \tau ]&= \frac{1}{2(t-\tau )}\exp \Big \{-\frac{S(t) + y}{2(t-\tau )}\Big \}\Big (\frac{S(t)}{y}\Big )^{\frac{n-2}{4}}\nonumber \\&\quad \Bigg [\Big (\frac{d}{dt}S(t) - \frac{S(t)}{t-\tau }+\mathcal {K}(t)\Big )\times I_{n/2-1}\Big (\frac{\sqrt{yS(t)}}{(t-\tau )}\Big )\nonumber \\&\quad + \frac{\sqrt{yS(t)}}{(t-\tau )} I_{n/2}\Big (\frac{\sqrt{yS(t)}}{(t-\tau )}\Big )\Bigg ], \end{aligned}$$

where $\mathcal {K}(t)$ is as:

14 $$\begin{aligned} \mathcal {K}(t) = \frac{1}{2} \Big (n - 1 - \frac{d}{dt}S(t)\Big ), \end{aligned}$$

This kernel function satisfies the following vanishing property as $\tau$ tends to *t* (Giorno et al., 1989):

15 $$\begin{aligned} \lim _{\tau \rightarrow t^{-}} \Psi [S(t), t| S(\tau ), \tau ] = 0. \end{aligned}$$

The solution of the integral equation (10) with kernel function as defined in equation (13) gives the first-passage time distribution of an *n*-dimensional zero-drift Wiener process (see Appendix B for the uniqueness of the solution of this equation). Then, we can put the first-passage time *g*(*t*) into equation (7) and obtain the joint probability distribution (Appendix A contains an example of how the Girsanov theorem works when using a time-dependent threshold). Intuitively, the only change with the fixed threshold case is that we use the threshold value in the final stopping points in all calculations of the Girsanov term. As we already mentioned in (7), the Girsanov change-of-measure term is a function of $\pmb {X}(T)$, which is the final stopping point. In the case of a fixed threshold, the final stopping point is equal to the threshold. However, the value $\pmb {X}(T)$ in the presence of a time-dependent threshold is equal to the threshold value at the final stopping point, which is *b*(*T*). Accordingly, to evaluate $\pmb {\mu }\cdot \textbf{X}(T)$ in the presence of a time-dependent threshold, we should use the following Cartesian representation of choice:

- $$X_1(T) = b(T) \cos (\varphi _1),$$
- $$X_2(T) = b(T) \sin (\varphi _1) \cos (\varphi _2)$$, $$~~~~~~\vdots $$
- $$X_{n-1}(T) = b(T) \sin (\varphi _1) \sin (\varphi _2) ~\dots ~ \sin (\varphi _{n-2})\cos (\varphi _{n-1}),$$
- $$X_{n}(T) = b(T) \sin (\varphi _1) \sin (\varphi _2) ~\dots ~ \sin (\varphi _{n-2})\sin (\varphi _{n-1}).$$

### Numerical approximation of the integral equation

Various numerical techniques have been developed in the scientific computing literature for approximating the solution of the Volterra integral equation (10) (e.g., Parand & Rad, 2012; Parand et al., 2022). However, since this equation is a linear integral equation and usually the approximation domain is not too large, methods based on discretization can approximate the solution very efficiently. The rectangle left-point integration scheme is the simplest and one of the most efficient discretizing schemes, which is also used frequently in psychology (e.g., Smith, 2000, 2023; Voskuilen et al., 2016). To evaluate the first-passage time distribution at $t_{i} = i\Delta t$, $i = 1, 2, ~\dots ~, K$ (maximum time step), where $\Delta t = \frac{T_{max}}{K}$, the rectangle left-point integration scheme gives the following approximation:^6^

16 $$\begin{aligned} \tilde{g}[S(\Delta t), \Delta t| y_0, 0] = - 2 \Psi [S(\Delta t), \Delta t | y_0, 0], \end{aligned}$$

17 $$\begin{aligned} \tilde{g}[S(i \Delta t), i \Delta t | y_0, 0] =&- 2 \Psi [S(i\Delta t), i\Delta t | y_0, 0] \\ &+ 2 \Delta t \sum _{j=1}^{i-1} \tilde{g}[S(j\Delta t), j \Delta | y_0, 0 ]\nonumber \\&\quad \Psi [S(i\Delta t), i \Delta t | S(j\Delta t), j \Delta t]. \nonumber \end{aligned}$$

The convergence of this scheme for the integral equation (10) is presented in Appendix B (also see theorem 4 in Buonocore et al., 1987). Therefore, by converging the $\Delta t$ to zero (i.e., $\Delta t \rightarrow 0$), this approximation scheme gives a better approximation of the first-passage time distribution. This theoretical support grants that the approximation scheme (16) and (17) gives a precise approximation of the first-passage time distribution. Figure 3 (Lower panel) illustrates an example of marginal response time distribution estimated using the proposed integral equation method.

## Simulation study: Method

### Simulation scenarios

To evaluate the integral equation method for parameter estimation with the HSDM, we conducted a parameter recovery study. Specifically, we assessed two-, three-, and four-dimensional HSDMs with either a constant or time-dependent threshold, and assessed the parameter recovery for a range of different numbers of trials. In all simulations, we fixed $\sigma = 1$ (i.e., the diffusion coefficient) and the starting point to be the origin (i.e., an unbiased starting point). We estimated the parameters of the HSDM through maximum likelihood estimation; specifically, we utilized the differential evolution optimization routine implemented in Scipy.^7^

For the HSDM with a constant threshold, we performed the parameter recovery with five different numbers of trials: 50, 100, 150, 200, and 250. For each number of trials, we sampled 300 random parameter sets from the following distributions:

$$b \sim \mathcal {U}[0.5, 6], ~~~~~~ t_0 \sim \mathcal {U}[0.1, 1], ~~~~~~ \mu _i \sim \mathcal {U}[-6, 6].$$

For the HSDM with a time-dependent threshold, we assumed a linear collapsing function for the decision threshold. Specifically, we selected the linear collapsing threshold based on good parameter recovery performance in the standard DDM (Evans et al., 2020), and the much poorer parameter recovery performance of nonlinear collapsing thresholds (Evans et al., 2020; Murrow & Holmes, 2024a). The linear dynamic for the decision threshold has also been utilized in several studies analyzing human behavior (e.g., Ashby, 1983; Diederich & Busemeyer, 2006; Katsimpokis et al., 2020). For the linear collapsing threshold, the decision threshold starts from $b_0$ and then decays linearly with the slope $\lambda$. In other words, $b(t) = b_0 - \lambda t$. Similar to the constant threshold model simulation, for each number of trials, we sampled 300 random parameter sets from the following distributions:

$$ b_0 \sim \mathcal {U}[2, 5], ~~~~~ \lambda \sim \mathcal {U}[0.1, 2], ~~~~~ t_0 \sim \mathcal {U}[0.1, 1], ~~~~~ \mu _i \sim \mathcal {U}[-3, 3]. $$

These distributions differ slightly from those used in the constant-threshold simulations. They were selected to ensure that the effect of the collapsing threshold is observable in the simulated data. For example, if the drift vector has a large magnitude, the evidence accumulation process reaches the decision threshold very quickly, and the effect of the collapse disappears from both response times and choices. Similarly, if the initial threshold value is very small, the threshold rapidly reaches the singular point (i.e., $b(t)=0$), and most decisions become random with very short response times – similar to fast guesses. Since the linear collapsing threshold model is more complex than the fixed threshold model, it may require additional trials to properly constrain the collapse. Therefore, we performed the parameter recovery with five different numbers of trials: two of which matched the constant thresholds simulation (100, 250), and three of which had a greater number of trials (500, 750, 1000).

### Evaluation metrics

To evaluate the quality of parameter estimation for the integral equation method, we utilized three different metrics. The first one is the root mean squared error (RMSE), which is defined as follows:

$$ RMSE = \sqrt{\frac{\sum _{i=1}^{N} (\theta _i - \hat{\theta }_i)^2}{N}}, $$

in which $\theta$ and $\hat{\theta }$ represent the actual (i.e., true) and estimated parameters, respectively, and *N* represents the number of data points. This metric assesses how accurately the method can recover the ground-truth parameter value. A lower value for this metric indicates a better parameter recovery.

**Fig. 4:**
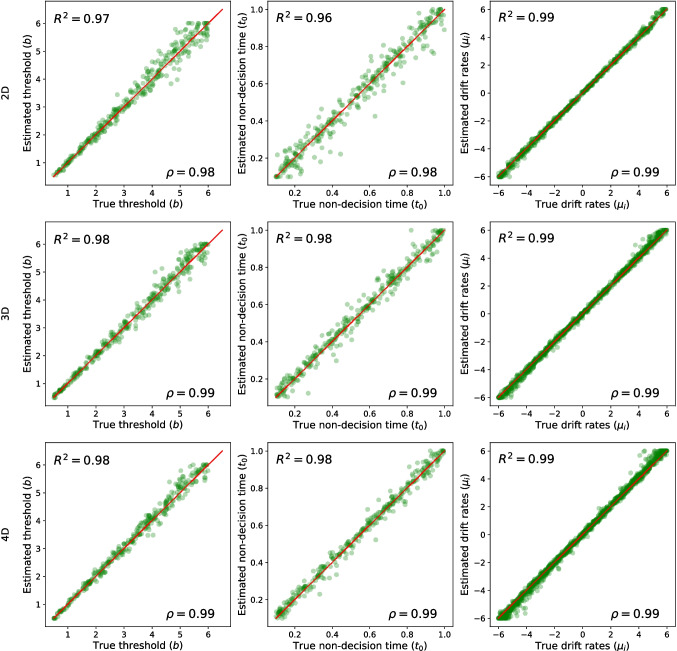
The parameter recovery plots for three HSDMs with a constant threshold. In each panel, the *x*-axis corresponds to the actual parameter value (true parameter), and the *y*-axis corresponds to the estimated parameter. The *first row* shows the parameter recovery of a two-dimensional HSDM (i.e., circular diffusion model). The second row presents the parameter recovery of the three-dimensional HSDM (i.e., spherical diffusion model), and the last row exhibits the parameter recovery of the four-dimensional HSDM

**Fig. 5:**
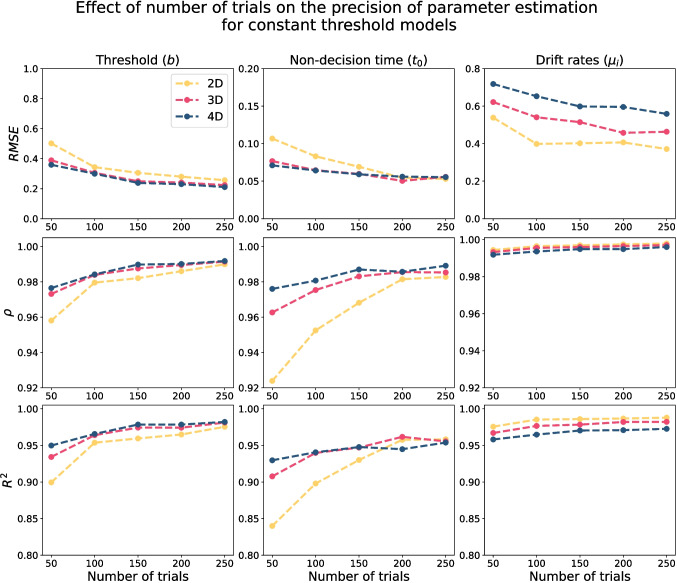
The quality of parameter estimation for constant threshold models based on the integral equation method (with $\Delta t = 0.05$) as a function of the number of trials. The *first row* shows the root mean square error (RMSE) for different numbers of trials (i.e., 50, 100, 150, 200, 250), the *second row* shows the correlation value ($\rho$), and the *third row* shows the R-squared value ($R^2$). Each column corresponds to one parameter, and *different colors* represent different models

The second metric is the correlation between the true parameters and estimated parameters, which is defined as follows:

$$\rho = \frac{\sum _{i=1}^{N}(\theta _i - \bar{\theta })(\hat{\theta }_i - \bar{\hat{\theta }})}{\sqrt{\sum _{i=1}^{N}(\theta _i - \bar{\theta })^2 \sum _{i=1}^{N}(\hat{\theta }_i - \bar{\hat{\theta }})^2}}, $$

where $\bar{\theta }$ and $\bar{\hat{\theta }}$ are mean values for the true parameters and estimated parameters, respectively. This metric evaluates the relation between the ground truth parameter and the estimated parameter.

The last metric is the R-squared ($R^2$):

$$R^2 = 1 - \frac{\sum _{i=1}^{N} (\theta _i - \hat{\theta }_i)^2}{\sum _{i=1}^{N}(\theta _i - \bar{\theta })^2}. $$

This metric compares the proportion of variance in the ground truth and estimated parameters. In other words, variability in parameter estimation results in a lower R-squared value. In general, these three metrics enable us to assess the parameter recovery performance in three slightly different ways, thereby providing a more comprehensive understanding of the integral equation method’s performance.

## Simulation study: Results

### Constant threshold models

The quality of parameter recovery based on 250 trials using the integral equation method (with $\Delta t = 0.02$) for the two-, three-, and four-dimensional HSDM with constant threshold is reported in Fig. 4.^8^ Importantly, these results seem to indicate that the parameter recovery of the HSDM using the proposed integral equation method is extremely accurate. Specifically, the R-squared ($R^2$) values for all parameters in all models are greater than 0.96, and correlation values ($\rho$) between the ground truth and estimated parameters are greater than 0.98, which reflects high parameter recovery precision. Furthermore, given the wide range of generating parameter values, particularly for the decision threshold (*b*) and the drift rate elements ($\mu _i$), these results confirm the stability of the integral equation method even for high values of threshold and drift length.

**Fig. 6:**
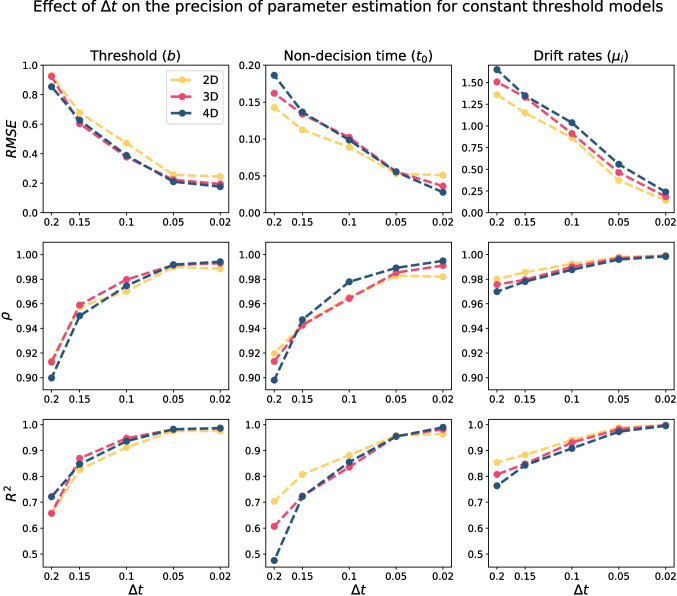
The convergence plot for parameter estimation based on the integral equation method for different $\Delta t$ values (i.e., 0.2, 0.15, 0.1, and 0.05). The *first row* shows the root mean square error (RMSE) as a function of $\Delta t$, the *second row* shows the correlation value ($\rho$), and the *third row* shows the R-squared value ($R^2$). Each *column* corresponds to one parameter, and *different colors* represent different models

The effect of the number of trials on the quality of parameter estimation (with $\Delta t = 0.05$) is illustrated in Fig. 5. As this figure shows, by increasing the number of trials, we have a more reliable estimation of the parameters. However, even with a very low number of trials (i.e., 50), the parameter recovery remains highly accurate for most parameters in most cases. Interestingly, it appears that the non-decision time and decision threshold parameters are more sensitive to the number of trials than the drift rates, with the non-decision time parameter in the two-dimensional model showing the greatest sensitivity to trials. However, regardless of the number of trials or the type of model, all parameters have very high correlations ($\rho > 0.9$) and R-squared values ($R^2 > 0.8$), showcasing accurate overall recovery performance.

**Fig. 7:**
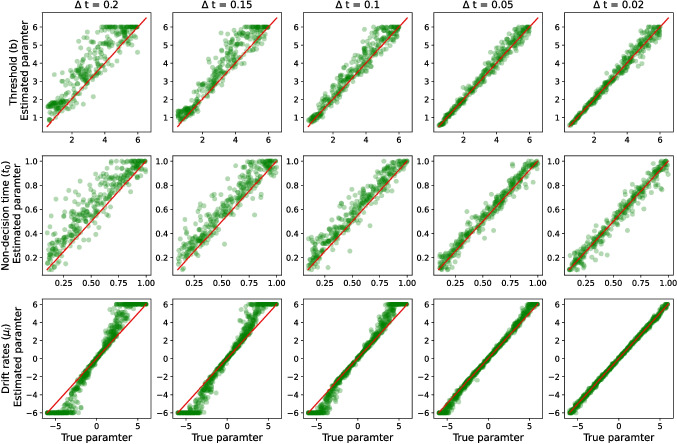
Illustration of the effect of $\Delta t$ on the precision of parameter recovery for the fixed threshold circular diffusion model. In each panel, the *x*-axis corresponds to the true generating parameter, and the *y*-axis corresponds to the estimated parameter

In addition to the number of trials, since the accuracy of the first-passage time approximation is associated with $\Delta t$, we also assessed the sensitivity of the parameter recovery to the $\Delta t$ value. Figure 6 illustrates how the RMSE, correlation ($\rho$), and r-squared ($R^2$) change as a function of $\Delta t$. In general, Figure 6 shows that the smaller the $\Delta t$ value, the better parameter estimation (at least down to $\Delta t = 0.02$), with RMSE, $\rho$, and $R^2$ all being systematically improved, and often by large margins, as $\Delta t$ becomes smaller. Another interesting result is the extreme sensitivity of the non-decision time estimation to the $\Delta t$ value, which is significantly greater than the other parameters. The reason behind this sensitivity is likely the approximation of the leading edge of the first-passage time distribution. Previous literature has shown that estimates of the non-decision time parameter are highly constrained by the leading edge of the first-passage time distribution (Ratcliff & Tuerlinckx, 2002). Importantly, by increasing the $\Delta t$ value in the utilized approximation scheme (16) and (17), the approximation of the leading edge of the first-passage time distribution becomes worse, which likely has a disproportionately large impact on the accuracy of the non-decision time estimate. Therefore, for a precise estimate of non-decision time, we recommend using $\Delta t \le 0.1$.

**Table 1:** The mean computational time (in milliseconds) for the integral equation method and the Bessel series method to compute the likelihood function for 250 trials

Integral equation				Series			
$\Delta t$	2D	3D	4D	N	2D	3D	4D
0.20	2.0	1.9	2.0	25	9.9	35.2	9.8
0.15	3.4	3.2	3.5	50	26.3	73.2	26.2
0.10	7.6	7.6	7.6	75	49.0	118.2	50.9
0.05	29.6	29.2	30.3	100	84.0	156.3	83.0

A more comprehensive view on the effect of $\Delta t$ on parameter estimation is presented by Fig. 7 (see also Figs. 13 and 14 for three- and four-dimensional models). This figure shows that for the high values of $\Delta t$ (particularly, $\Delta t \ge 0.1$), there is a systematic overestimation in threshold and non-decision time. Similarly, the drift rate is overestimated/underestimated for high/low drift values when the $\Delta t \ge 0.1$. These results are also replicated for three- and four-dimensional HSDM (see Appendix D). Figures 6 and 7 suggest that as $\Delta t$ becomes smaller, the systematic biases in parameter estimation disappear. The results reported by Richter et al. (2023) also confirm that $\Delta t$ around 0.05 to 0.02 is sufficient for parameter estimation of the fixed threshold model, and there is no need for a smaller value of $\Delta t$.

**Fig. 8:**
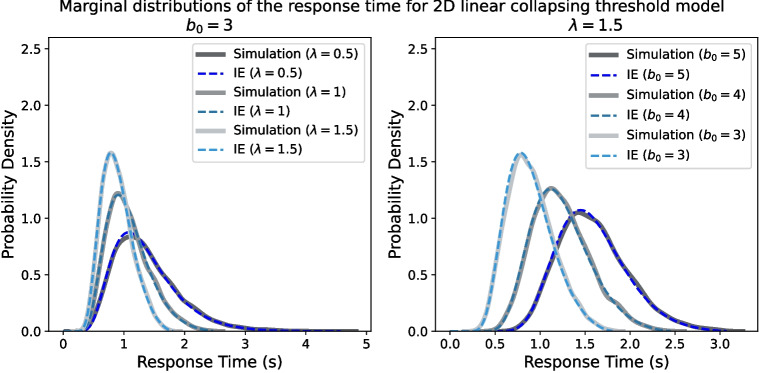
The marginal distribution of response time for 10,000 simulated trajectories with $\pmb {\mu }=[1, 1]'$, and linear collapsing threshold in two-dimensional HSDM (i.e., linear collapsing circular diffusion model). The *left panel* shows the response time distribution with $b_0=3$ and different slopes. The *right panel* shows the response time distribution for $\lambda =1.5$ and different starting thresholds. The *solid gray lines* represent the kernel density estimation for the simulated data, while the *blue dashed lines* denote the predictions obtained using the integral equation method

It is worth noting that there is a tradeoff between the precision of the first-passage time estimation and the computation time. In other words, by using smaller values of $\Delta t$, the approximation scheme (17) gives a more accurate estimation of the first-passage time distribution, but at the cost of a longer time to estimate. Table 1 presents how $\Delta t$ can affect the computational time for our proposed integral equation method compared to the existing Bessel series method. While using smaller values of $\Delta t$ can substantially increase the computation time for the integral equation method – particularly in the case of $\Delta t = 0.05$ – this is also the case for the Bessel series method, with the computation time of the integral equation method always being substantially (i.e., minimum 2.5 times) shorter than the Bessel series method. The other interesting pattern regarding computational time is that the number of dimensions of the accumulation process does not impact the computational time in the integral equation method, though the computational time of the Bessel series becomes substantially longer for the three-dimensional model (spherical diffusion model).^9^

**Fig. 9:**
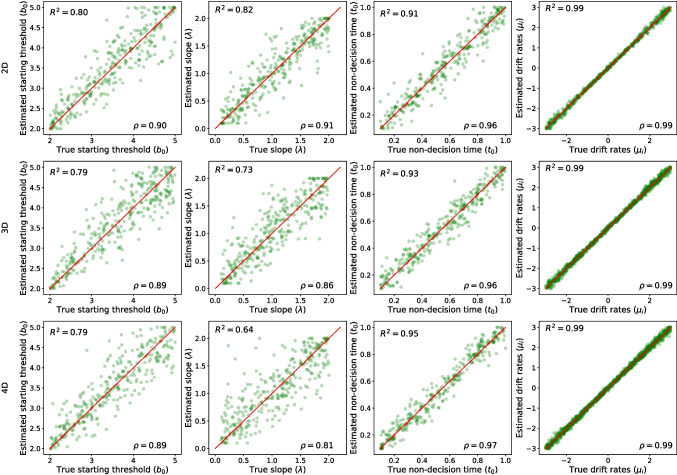
The parameter recovery plots for three HSDMs with the linear collapsing threshold. In each panel, the *x*-axis corresponds to the actual parameter value (true parameter), and the *y*-axis corresponds to the estimated parameter. The *first row* shows the parameter recovery of a two-dimensional HSDM (i.e., circular diffusion model). The *second row* presents the parameter recovery of the three-dimensional HSDM (i.e., spherical diffusion model), and the *last row* exhibits the parameter recovery of the four-dimensional HSDM. All parameters were being estimated simultaneously

As an additional assessment, we also performed a parameter recovery with across-trial variability in the drift rate included in the model, which is reported in the Appendix E. Interestingly, the main parameters of the model are still recovered very precisely, though the estimation of the variability parameter exhibits somewhat higher uncertainty, which is consistent with observations in the standard DDM (Boehm et al., 2018). Overall, our results for the HSDMs with a constant threshold suggest that the integral equation method can reliably and efficiently estimate the parameters of the model, and do so with a shorter computation time than the existing and less accurate Bessel series method.

### Time-dependent threshold models

First, as a proof of concept to ensure that the integral equation method provides a reasonably accurate approximation of the likelihood function for the collapsing threshold model, we looked at how closely the likelihood function of the integral equation method matches data simulated from the models. Figure 8 illustrates the marginal response time distribution for six examples of linear collapsing circular diffusion models. For each parameter value, 10,000 trials are simulated from the model, and a kernel density estimation of the response time distribution is used to create a pseudo-likelihood function. In addition to linear collapsing models, we have also tested for nonlinear collapsing models, and the results are available in Appendix F. Importantly, the match between the model simulations and the likelihood function of the integral equation method is extremely close, showing a proof-of-concept level success of the method.

Figure 9 illustrates the quality of parameter recovery based on 500 trials for the linear collapsing HSDMs using the integral equation method with $$\Delta t = 0.02$$. Interestingly, the drift rate parameter is recovered extremely well across all models, which is consistent with results for the linear collapsing DDM (Evans et al., 2020). All other parameters of two- and three-dimensional HSDM are recovered reasonably well. However, it should be noted that the starting threshold in the linear collapsing model is recovered more poorly than the decision threshold in the constant thresholds model, again consistent with results for the linear collapsing DDM (Evans et al., 2020). However, the slope parameter in the four-dimensional HSDM shows quite a large variability in estimation, with a general trend of the slope parameter becoming increasingly difficult to estimate as the number of dimensions of the model increases (i.e., $$R^2 = 0.82, 0.73, $$ and 0.64 for two-, three- and four-dimensional linear collapsing HSDM, respectively).

**Fig. 10:**
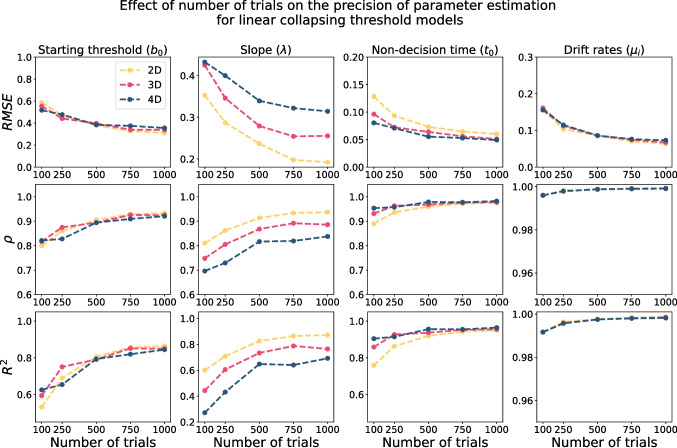
The quality of parameter estimation for linear collapsing threshold models based on the integral equation method (with $\Delta t = 0.02$) as a function of the number of trials. The *first row* shows the root mean square error (RMSE) as a function of $\Delta t$, the *second row* shows the correlation value ($\rho$), and the *third row* shows the R-squared value ($R^2$). Each *column* corresponds to one parameter, and *different colors* represent different models

Similar to constant threshold models, we also checked the sensitivity of the parameter estimation of collapsing models to the number of trials and the value of $\Delta t$. Figure 10 illustrates the precision of parameter recovery (with $\Delta t = 0.02$) as a function of the number trials: 100, 250, 500, 750, and 1000. The general pattern is more precise estimation as the number of trials increases, with the most sensitive parameters being the starting threshold ($b_0$) and the slope of collapse ($\lambda$). The results suggest that to have a reasonably reliable estimation of the starting threshold and slope parameters, at least 500 trials are required. However, the non-decision time and drift rate parameters are less sensitive to the number of trials, and can be estimated reliably even based on 250 trials.

**Fig. 11:**
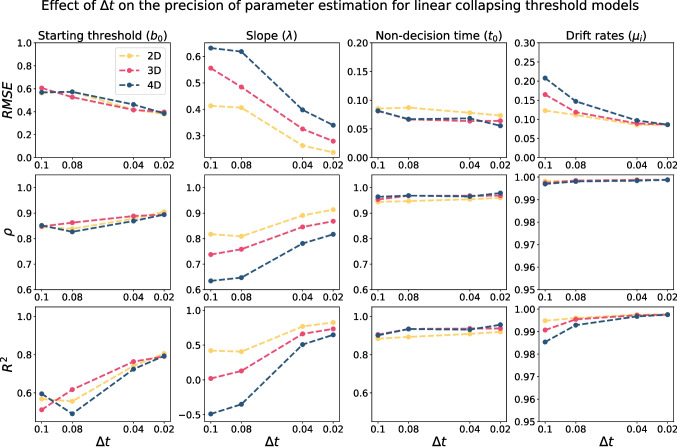
The quality of parameter estimation for linear collapsing threshold models based on 500 trials using the integral equation method as a function of $\Delta t$. The *first row* shows the root mean square error (RMSE) as a function of $\Delta t$, the *second row* shows the correlation value ($\rho$), and the *third row* shows the R-squared value ($R^2$). Each column corresponds to one parameter, and different colors represent different models

**Fig. 13:**
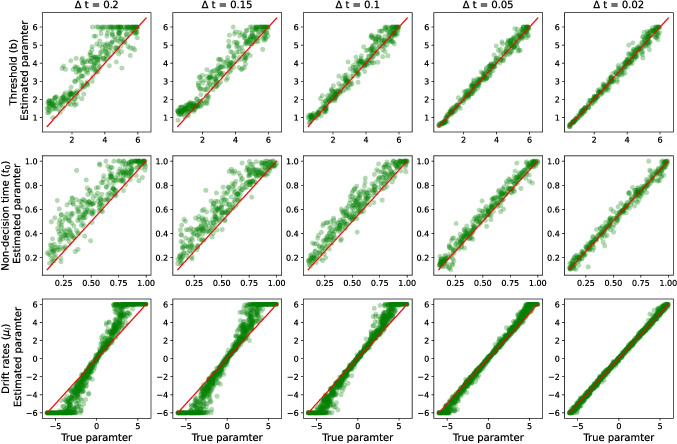
Illustration of the effect of $\Delta t$ on the precision of parameter recovery for the fixed threshold spherical diffusion model. In each panel, the *x*-axis corresponds to the true generating parameter, and the *y*-axis corresponds to the estimated parameter

**Fig. 14:**
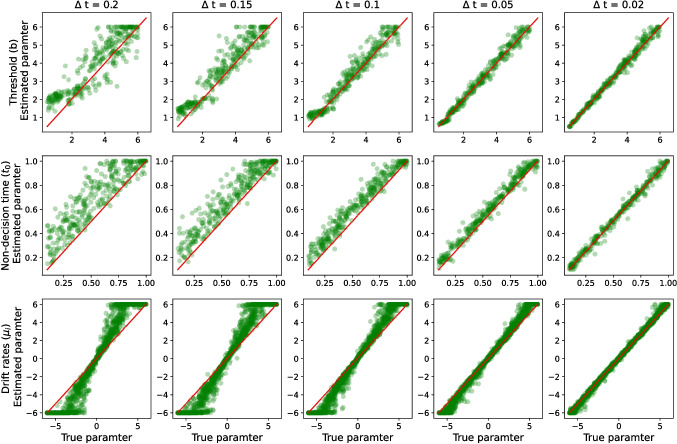
The illustration of the effect of $\Delta t$ on the precision of parameter recovery for the fixed threshold hyper-spherical diffusion model. In each panel, the *x*-axis corresponds to the true generating parameter, and the *y*-axis corresponds to the estimated parameter

Figure 11 illustrates the effect of $\Delta t$ on the precision of parameter recovery of collapsing threshold models. In general, to have reliable parameter estimation for collapsing threshold models, a smaller $\Delta t$ is required compared to the constant threshold models. For instance, even $\Delta t = 0.1$ gives a reasonably good estimation of parameters in constant threshold models, though it provides an extremely poor estimation of the slope parameter in the linear collapsing thresholds models.

## General discussion

**Fig. 16:**
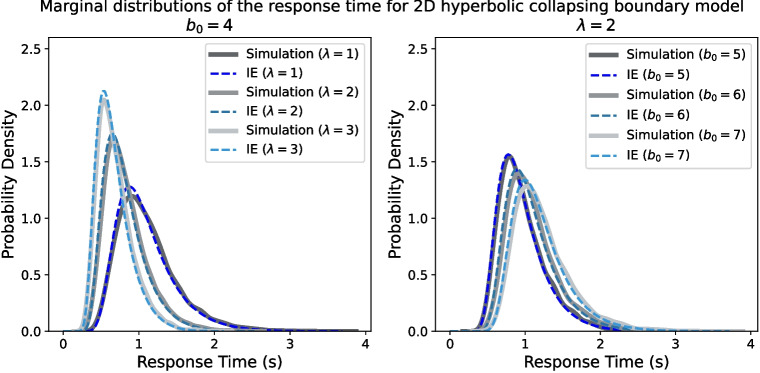
The marginal distribution of response time for $10\,000$ simulated trajectories with $\pmb {\mu }=(1, 1)$, and hyperbolic collapsing threshold in two-dimensional HSDM (i.e., hyperbolic collapsing circular diffusion model). The *left panel* shows the response time distribution with $b_0=4$ and different decay rates. The *right panel* shows the response time distribution for $\lambda =2$ and different starting thresholds. The *solid gray lines* represent the kernel density estimation for the simulated data, while the *blue dashed lines* denote the predictions obtained using the integral equation method

**Fig. 17:**
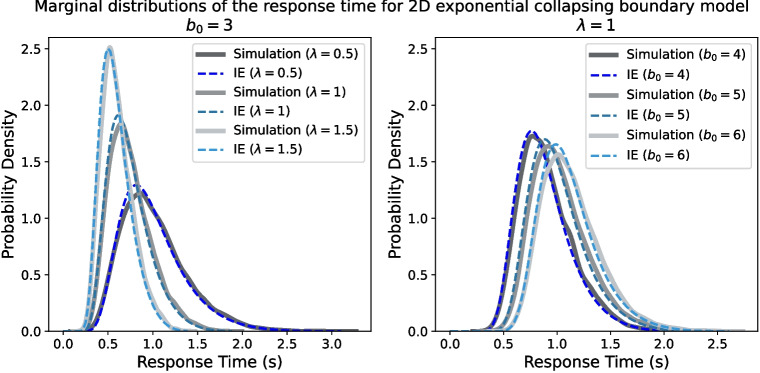
The marginal distribution of response time for $10\,000$ simulated trajectories with $\pmb {\mu }=(1, 1)$, and exponential collapsing threshold in two-dimensional HSDM (i.e., exponential collapsing circular diffusion model). The *left panel* shows the response time distribution with $b_0=3$ and different decay rates. The *right panel *shows the response time distribution for $\lambda =1$ and different starting thresholds. The *solid gray lines* represent the kernel density estimation for the simulated data, while the *blue dashed lines* denote the predictions obtained using the integral equation method

Understanding whether people adjust their decision threshold over the time course of a single decision is one of the central topics that have been studied in economics (e.g., Fudenberg et al., 2018, 2020; Olschewski et al., 2025), neuroscience (e.g., Drugowitsch et al., 2012; Murphy et al., 2016), and cognitive psychology (Evans & Hawkins, 2019; Evans et al., 2020; Smith & Ratcliff, 2022) for many years. However, most of these studies have focused on binary choice decisions, with more complex continuous response decisions being largely ignored due to the lack of methodology for estimating time-dependent parameters in continuous response models. This paper developed a methodology that allows for a greater integration of these separate research areas, where we considered the HSDM – a SSM model of decisions with continuous response scales – and introduced an analytical solution for the time-varying threshold HSDM. More specifically, we proposed an integral equation method to estimate the parameters of HSDM with a constant or time-dependent threshold. This relies on transforming the Bessel process into a Feller process and then using the kernel function of the Feller process for the proposed integral equation. The solution of the integral equation gives the first-passage time distribution of the zero-drift process in the presence of a constant/time-dependent threshold. Then, by applying the Girsanov change-of-measure theorem – which was also utilized by Smith (2016) for constant threshold diffusion model – we obtain the joint distribution of the choice and response time of the non-zero drift process.^10^ Our proof of concept simulation results (see Figs. 8, 16, and 17) also showcased the general accuracy of the method, confirming the applicability of the Girsanov change-of-measure theorem for time-dependent threshold models.

The proposed estimation method has several advantages over other existing methods. First, there is a theoretical guarantee that the solution of the integral equation converges to the first-passage time distribution of the considered process. Therefore, by considering a smaller $\Delta t$, a more precise estimation of the first-passage time distribution can be obtained. In practice, for constant threshold models, there is no need to consider very small $\Delta t$, as even with coarse-grained $\Delta t$ the method provides an accurate approximation of the first-passage time distribution. However, for time-dependent threshold models, small values of $\Delta t$ are required, as larger values lead to inaccuracies. The second advantage is the lower computational cost. Approximating the solution of the integral equation based on the rectangle left-point scheme is relatively fast, and the method does not need any costly computations, making it considerably faster than the Bessel series method. The third advantage is the flexibility of the method, as the integral equation method can be utilized for both constant or collapsing-threshold models, provided that the collapse follows a specific parametric function. In addition to these theoretical and computational benefits, the parameter recovery results showed that the parameter estimation based on the integral equation method is very reliable and provides an accurate parameter recovery of HSDMs with a range of dimensions.

The other important aspect of the results was the effect of the number of trials on the precision of parameter recovery. These results can help ensure that empirical studies aiming to use these models are designed with sufficient trials for reliable estimation. Figure 5 suggests that, even based on 50 trials, the parameters of the constant threshold HSDM can be estimated reasonably reliably. However, most parameters show even greater recovery with 100 trials, with the recovery continuing to improve up to at least 200 trials. Therefore, we recommend that researchers aim to collect at least 100–200 trials when possible, in order to maximize the reliability of the parameter estimation for constant threshold HSDM, and to help ensure the stability of other behavioral patterns. In contrast, a large number of trials are required to even reasonably reliably estimate the parameters of the collapsing threshold HSDM. Figure 10 suggests that at least 500 trials are required for even reasonable parameter recovery of all parameters of the collapsing threshold HSDM, with the slope parameter being particularly difficult to estimate.

It is also worth highlighting that the Feller process has previously been used for the linear drift linear infinitesimal variance (LDLIV) model (Smith, 2023), where the general form of the Feller process, which also includes leakage, can represent a biologically plausible model of decision making. Furthermore, Smith (2023) also proposed an integral equation for the likelihood estimation of the LDLIV model, with the integral equation in this paper being a special case of the integral equation that is proposed for LDLIV in Smith (2023). Since the HSDM and LDLIV models (or, more generally, the Feller process and Bessel process) are connected, by proposing a method for one model, it can also be utilized for the other model.

### Comparing with other estimation methods

One of the competing methods for estimating the first-passage time is the partial differential equation (PDE) method. This method represents the first-passage time or transition probability distribution of a Wiener process as the solution of a Fokker–Planck equation. This method has been utilized in psychology for estimating the parameters of the diffusion decision models with constant (Hadian Rasanan et al., 2024; Voss & Voss, 2008) or time-dependent parameters (Boehm et al., 2021; Murrow & Holmes, 2024b; Hadian Rasanan et al., 2023; Richter et al., 2023; Shinn et al., 2020). Although this method is very powerful, flexible, and efficient for parameter estimation of models of binary-choice tasks, it is not suitable for the parameter estimation of HSDM. The corresponding Fokker–Planck equation for an *n*-dimensional Wiener process is an *n*-dimensional equation, and approximating the solution of such a high-dimensional equation is challenging for numerical algorithms. In other words, by increasing the dimensionality of the accumulation process in HSDM, the dimensionality of the corresponding Fokker–Planck equation also increases, and it makes the estimation of the solution harder. For instance, to estimate the parameters of four-dimensional HSDM (Smith & Corbett, 2019), one would need to estimate the solution of a four-dimensional Fokker–Planck equation, which can be very time-consuming and demanding (see Appendix G for a mathematical discussion on the PDE method). In contrast with the PDE method, the dimensionality of the integral equation proposed in this paper would not change, and as shown in this article, there is a one-dimensional Volterra integral equation for HSDM with any dimension. Furthermore, it should be noted that the results of Richter et al. (2023) showed that the integral equation method is relatively precise in parameter estimation of diffusion models and has the same level of precision in parameter recovery compared to the PDE method. One could also argue that instead of using the *n*-dimensional Fokker–Planck equation corresponding to process (2), it would be possible to use the one-dimensional Fokker–Planck equation (Sacerdote, 1990) corresponding to (8). While this approach can solve the problem of dimensionality, it still has some complexity in approximating the solution (e.g., handling the Dirac-delta function as the initial condition and variable coefficient), and the integral equation method is a much more straightforward approach.

A more general technique that can be utilized for parameter estimation of complex models is the probability density approximation (PDA) methods (e.g., chandrasekaran & Hawkens, 2019; Holmes, 2015; Turner & Sederberg, 2014). Generally, the PDA approach works by using a large number of simulated trials to approximate the likelihood function through kernel density estimation methods. This method is beneficial when the model is less mathematically tractable, and other analytical techniques to provide an efficient estimate of the likelihood function are not available. While the PDA methods are very flexible and can be utilized for parameter estimation of any model, they can be imprecise with smaller numbers of simulated trials, and time-consuming with a sufficiently large number of simulated trials to achieve high precision^11^, meaning that they always need to be tailored and tested for specific models and situations to ensure sufficient reliability of estimates.

Recently, the neural network approaches for parameter estimation have provided an important alternative approach to PDA methods (e.g., Ghaderi-Kangavari et al., 2023; Hadian Rasanan et al., 2024; Kvam et al., 2024; Schumacher et al., 2023; Sokratous et al., 2023). Similar to traditional PDA approaches, the neural network methods use a large number of simulated trials. However, in neural network approaches, the simulations are used to learn the model’s behavior, where deep neural network learns (approximates) the likelihood function (Fengler et al., 2021), the posterior distribution of the parameters (Radev et al., 2020), or both simultaneously (Radev et al., 2023), often allowing for more efficient parameter estimation than PDA methods. One of the main limitations of the neural network methods is the necessity for training for every new empirical data set, which can be very time-consuming, varying from several hours to several days depending on the model’s complexity. However, the integral equation method does not need massive simulations and training, making it a more efficient approach for models for which the integral equation is available. Another key limitation is that neural network approaches can only estimate the likelihood function within the parameter range for which the network was trained, and estimating a parameter outside this range can be challenging. Therefore, neural networks are dependent on priors considered for simulating the model’s behavior; a limitation that is not present when estimating the likelihood function based on the integral equation method.

### Limitations of the integral equation method

Although the proposed integral equation method performed well in estimating the parameters of both the constant and collapsing threshold HSDM, it still has some limitations. As previously mentioned, a more general version of the HSDM is the hyperelliptic diffusion model, which features an asymmetric decision threshold (Smith, 2016). In such a generalization, the threshold dynamics can also be asymmetric – an aspect not considered in the present work. For example, the threshold may collapse more rapidly along the *y*-axis than along the *x*-axis, resulting in a decision boundary that becomes increasingly elliptical over time. A model with asymmetric threshold dynamics may be useful for capturing human behavior in environments with external biases. For instance, if participants are aware that the correct response is located at 0 or 180 degrees in $70\%$ of the trials, then random decisions are more likely to cluster around those directions, which could be reflected by a higher collapsing rate along the *x*-axis. For this class of more complex models, simulation-based methods remain necessary.

### Future directions

In this work, we only considered the maximum likelihood procedure for estimating the parameters of the HSDM. How-ever, one could be interested in Bayesian parameter estimation techniques, which can also provide an estimate of the uncertainty in the estimated parameter values.^12^ Importantly, previous work has combined likelihood approximation techniques like PDE (Murrow & Holmes, 2024b), PDA (Evans & Servant, 2020, 2022; Gunawan et al., 2020; Turner et al., 2013), or neural network methods (Radev et al., 2020) with Bayesian inference techniques. Of particular note, the computational package PyBeam (Murrow & Holmes, 2024b) provides a user interface for parameter estimation of computational models of binary choice tasks using the PDE method with Bayesian inference, providing a user-friendly method for performing Bayesian inference on complex models. Therefore, a natural next step would be to combine the integral equation method with Bayesian parameter estimation techniques and to develop a (Python or R) package to make the HSDM more accessible to empirical researchers analyzing human behavior from continuous-response tasks. However, it is worth mentioning that there is already a computational package called JAGS-CDDM, which provides a Bayesian parameter estimation framework for the circular diffusion model with constant threshold (Villarreal et al., 2024). However, it is limited only to the circular diffusion model (two-dimensional HSDM) and does not support higher-dimensional HSDMs or time-varying threshold models.

Finally, the focus of this paper was not to propose or test a new model. However, an interesting future direction would be to investigate the collapsing threshold mechanism for continuous-response tasks, particularly given the mixed findings in binary response tasks (Evans & Hawkins, 2019; Evans et al., 2020; Hawkins et al., 2015; Milosavljevic et al., 2010; Olschewski et al., 2025; Smith & Ratcliff, 2022; Voskuilen et al., 2016). The collapsing HSDM considered in this paper, along with the integral equation method for parameter estimation of this model, provides an easy-to-use modeling framework to test the collapsing threshold mechanism in continuous-response tasks. Given that some research has suggested that people only use collapsing thresholds in certain situations (Evans & Hawkins, 2019; Evans et al., 2020; Hawkins et al., 2015), our integral equation method can help improve our understanding of how and in which situations people use collapsing thresholds by allowing the potential situations to also include tasks with a continuous range of options. Answering these questions can be very beneficial for a deeper insight into the underlying cognitive processes of human decisions in many situations, especially for exploring whether people behave optimally in continuous response tasks.

### Conclusion

Despite recent advances in computational models for conti-nuous-response decisions, these models have some challenges in parameter estimation. The present work considered the hyper-spherical diffusion models with constant and time-dependent thresholds and proposed an integral equation method for parameter estimation. To evaluate the method, we have conducted a parameter recovery study for models with constant or time-dependent thresholds. The results demonstrated that the integral equation method is relatively fast, accurate, and reliable in estimating the parameters of the hyper-spherical diffusion models.

## Figures and Tables

**Fig. 12:**
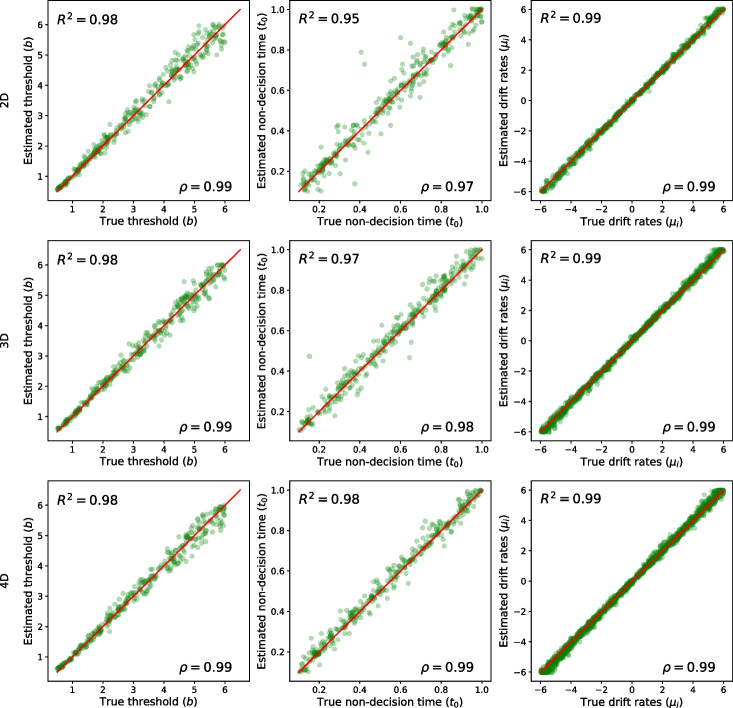
The parameter recovery plots based on the Bessel series method for three HSDMs with a constant threshold. In each panel, the *x*-axis corresponds to the actual parameter value (true parameter), and the *y*-axis corresponds to the estimated parameter. The *first row* shows the parameter recovery of a two-dimensional HSDM (i.e., circular diffusion model). The *second row* presents the parameter recovery of the three-dimensional HSDM (i.e., spherical diffusion model), and the *last row* exhibits the parameter recovery of the four-dimensional HSDM

**Fig. 15:**
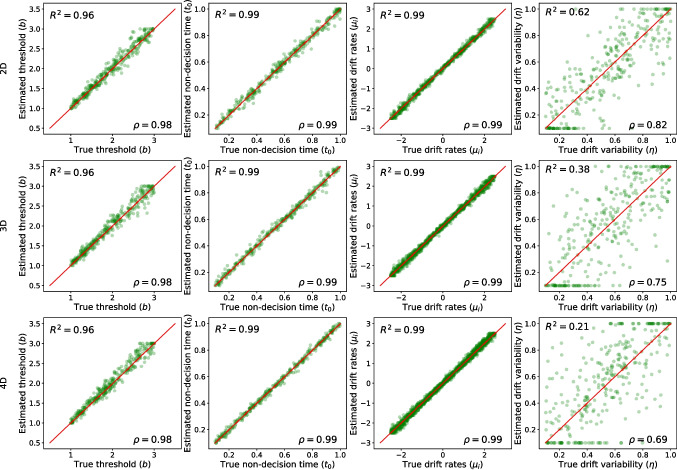
The parameter recovery plots for three HSDMs with constant boundary in the presence of across-trial variability in drift rate. In each panel, the *x*-axis corresponds to the actual parameter value (true parameter), and the *y*-axis corresponds to the estimated parameter. The *first row* shows the parameter recovery of a two-dimensional HSDM (i.e., circular diffusion model). The *second row* presents the parameter recovery of the three-dimensional HSDM (i.e., spherical diffusion model), and the *last row* exhibits the parameter recovery of the four-dimensional HSDM

## Data Availability

All codes and data supporting this paper are publicly available on: https://github.com/AmirHoseinHadian/IE4HSDM

## Appendix A The Girsanov theorem

This appendix aims to present an intuition about the Girsanov change-of-measure theorem (Girsanov, 1960) and present an example of its application to processes in the presence of a time-dependent absorbing threshold. However, interested readers can refer to Øksendal (2013) or Karatzas and Shreve (2014) for a more rigorous discussion. Consider the following Ito$\hat{\text {p}}$rocess with a general drift rate $\mu \big (X(t), t\big )$, which starts from 0:

18 $$\begin{aligned} dX(t) = \mu \big (X(t), t\big ) dt + dW(t), ~~~~~ X(0) = 0, \end{aligned}$$

The Girsanov change-of-measure theorem states that for any bounded measurable function *h*, the expectation of $h\big (X(t)\big )$ can be obtained as follows:

19 $$\begin{aligned} \mathbb {E}[h\big (X(t)\big )] = \mathbb {E}[M(t) h\big (W(t)\big )], \end{aligned}$$

in which *M*(*T*) is defined as below:

20 $$\begin{aligned} M(T) = \exp \Big [ \int _0^T \mu \big (W(s), s\big ) dW(s) - \frac{1}{2} \int _0^T \mu ^2\big ( W(s), s\big ) ds\Big ]. \end{aligned}$$

In simple words, if we have a measurement of the zero-drift process, we can extend it to the non-zero drift process by multiplying *M*(*t*) by the measurement of the zero-drift process. In the case of Bessel process, we have a *n*-dimensional Wiener process with constant drift vector $\pmb {\mu } = [\mu _1, \dots , \mu _n]'$. Thus, *M*(*t*) is obtained as follows:

21 $$\begin{aligned} M(T)= &  \exp \Big [\sum _{i=1}^{n} \int _0^T \mu _i dW(s) - \frac{1}{2} \int _0^T \mu _i^2 ds\Big ] \nonumber \\= &  \exp \Big [\pmb {\mu }\cdot \textbf{X}(T) - \frac{1}{2}\Vert \pmb {\mu }\Vert ^2 T\Big ]. \end{aligned}$$

Also, if the diffusion coefficient is not equal to one, then *M*(*T*) becomes as follows:

22 $$\begin{aligned} M(T) = \exp \Big [\frac{1}{\sigma ^2}\pmb {\mu }\cdot \textbf{X}(T) - \frac{1}{2\sigma ^2}\Vert \pmb {\mu }\Vert ^2 T\Big ]. \end{aligned}$$

To obtain *M*(*T*), we need to determine the value of the final stopping point $\pmb {X}(T)$. In the case of a constant threshold model, $\pmb {X}(T)$ becomes equal to the threshold value. However, in the presence of a time-dependent threshold, the value of $\pmb {X}(T)$ is equal to the threshold value at the final stopping time (i.e., $\pmb {X}(T) = b(T)$). Smith (2016) illustrated how the Girsanov theorem can be applied to constant threshold diffusion models. Here, to illustrate how the Girsanov theorem can be utilized in situations where we have a time-dependent threshold, we will present an example of applying this theorem to the single-boundary Wiener process with a time-dependent threshold. The first-passage time distribution of a single-boundary Wiener process starting from zero (i.e., $x_0=0$) in the presence of a linear collapsing threshold (i.e., $b(t) = b_0 - \lambda t$) is as follows (Buonocore et al., 1987):

23 $$\begin{aligned} g(b_0 - \lambda t, t|0, 0) = \frac{b_0}{t} \frac{1}{\sqrt{2 \pi \sigma ^2 t }}\exp \Bigg (-\frac{\big (b_0 - \lambda t - \mu t\big )^2}{2 \sigma ^2 t}\Bigg ). \end{aligned}$$

If the first-passage time distribution of the zero-drift process is as below:

24 $$\begin{aligned} g_z(b_0 - \lambda t, t|0, 0) = \frac{b_0}{t} \frac{1}{\sqrt{2 \pi \sigma ^2 t }}\exp \Bigg (-\frac{\big (b_0 - \lambda t)^2}{2 \sigma ^2 t}\Bigg ), \end{aligned}$$

and *M*(*t*) be:

25 $$\begin{aligned} M(t) = \exp \Big [\frac{\mu b(t)}{\sigma ^2} - \frac{1}{2 \sigma ^2}\mu ^2 t\Big ], \end{aligned}$$

then, we will show that $g(b_0 - \lambda t, t|0, 0) = M(t) \times g_z(b_0 - \lambda t, t|0, 0)$. For simplicity, instead of $b_0 - \lambda t$, we just write *b*(*t*). So, we have:

26 $$\begin{aligned} M(t) \times g_z\big (b(t), t|0, 0\big )&= \exp (\frac{\mu b(t)}{\sigma ^2} - \frac{1}{2 \sigma ^2}\mu ^2 t)\\&\quad \times \frac{b_0}{t} \frac{1}{\sqrt{2 \pi \sigma ^2 t }}\exp \Bigg (-\frac{b^2(t)}{2 \sigma ^2 t}\Bigg )\nonumber \\&= \frac{b_0}{t} \frac{1}{\sqrt{2 \pi \sigma ^2 t }}\exp \Bigg (-\frac{b^2(t)}{2 \sigma ^2 t}+ \frac{\mu b(t)}{\sigma ^2} \nonumber \\&\quad - \frac{1}{2 \sigma ^2}\mu ^2 t\Bigg )\nonumber \\&= \frac{b_0}{t} \frac{1}{\sqrt{2 \pi \sigma ^2 t }}\exp \Bigg (-\frac{b^2(t)}{2 \sigma ^2 t} + \frac{2\mu b(t)t}{2\sigma ^2t} \nonumber \\&\quad - \frac{\mu ^2 t^2}{2 \sigma ^2 t}\Bigg )\nonumber \\&= \frac{b_0}{t} \frac{1}{\sqrt{2 \pi \sigma ^2 t }}\nonumber \\&\quad \exp \Bigg (-\frac{b^2(t) - 2\mu b(t)t + \mu ^2 t^2}{2 \sigma ^2 t}\Bigg )\nonumber \\&= \frac{b_0}{t} \frac{1}{\sqrt{2 \pi \sigma ^2 t }}\exp \Bigg (-\frac{\big (b(t) - \mu t\big )^2 }{2 \sigma ^2 t}\Bigg )\nonumber \\&= \frac{b_0}{t} \frac{1}{\sqrt{2 \pi \sigma ^2 t }}\exp \Bigg (-\frac{\big (b_0 - \lambda t - \mu t\big )^2}{2 \sigma ^2 t}\Bigg ) \nonumber \\&\quad = g\big (b(t), t|0, 0\big ).\nonumber \end{aligned}$$

So, in a very simple example, we have shown that the Girsanov change-of-measure theorem can also be applied to time-dependent threshold models. The only thing that should be considered is that in the presence of a time-dependent threshold, *X*(*T*) in the Girsanov formula becomes equal to the final value of the threshold *b*(*T*).

## Appendix B Theoretical support of volterra integral equation

This appendix contains some theoretical support for the proposed integral equation method in this paper. Mainly, Theorem B.2 shows that the integral equation (10) with the kernel function (13) has a solution, and this solution is unique. Moreover, Theorem B.3 shows that the considered approximation scheme (17) converges to the exact solution of the integral equation.

### Definition B.1

Let $\Omega$ be a subset of $\mathbb {R}^n$ and $f: \Omega \rightarrow \mathbb {R}$ be a function. Then *f* is Lipschitz on $\Omega$ if there exists a non-negative $L_f$ such that

$$\Vert f(x) - f(y) \Vert \le L_f \Vert x - y \Vert ,$$

for all $\textbf{x}, \textbf{y} \in \Omega$ . The real number $L_f$ is named the Lipschitz constant of *f*. The norm used here is the second-norm, defined as:

$$\Vert h \Vert = \left( \int _{\Omega } | h(\textbf{x}) |^2 \textbf{dx} \right) ^{1/2}$$

and

$$\Vert \textbf{x} \Vert = \left( x_1^2 + x_2^2 +... + x_n^2 \right) ^{1/2},$$

when *h* is a Lipschitz function and $\textbf{x} = [x_1, x_2,..., x_n]'$ is a vector in $\mathbb {R}^d$.

### Theorem B.1

(**Existence and Uniqueness of the Solution; ** Hackbusch & Hackbusch, 1995) Consider the following general form of the nonlinear Volterra integral equation of the second kind, which is defined on $\Omega$:

27 $$\begin{aligned} g(t) = h(t) + \int _{t_0}^{t} \Theta \big (t, \tau , g(\tau )\big ) d\tau . \end{aligned}$$

If there exists a function $L(t, \tau )$ such that the kernel function satisfies the following Lipschitz condition:

28 $$\begin{aligned} \big |\Theta \big (t, \tau , s'\big ) - \Theta \big (t, \tau , s''\big )\big | \le L(t, \tau ) \big |s' - s''\big |, \end{aligned}$$

then, there is exactly one solution $g \in C(\Omega )$ for the integral equation (27), where $C(\Omega ) = \{f: f ~is ~a ~continuous ~function ~on ~\Omega \}$.

### Proof

The proof is presented in Hackbusch and Hackbusch (1995). $\square$

### Theorem B.2

The integral equation (10) with the kernel function (13) has a unique solution.

### Proof

Since the integral equation (10) is a convolutional type integral equation, by considering $L(t, \tau ) = \big |\Psi [S(t), t| S(\tau ), \tau ]\big |$, the kernel function (13) satisfies the Lipschitz condition (28) and then, Theorem B.1 holds for the integral equation (10). $\square$

### Lemma B.1

(**Grönwall’s Inequality;** Quarteroni & Valli, 2008) Assume that $\gamma _n$ is a non-negative sequence and that the sequence $\alpha _n$ satisfies

$$\left\{ \begin{array}{ll} \alpha _0 \le \beta _0,  \alpha _n \le \beta _0 + \sum _{k=0}^{n-1} \varrho _k + \sum _{k=0}^{n-1} \gamma _k \alpha _k, ~~~~ n>0 \end{array}\right. $$

then $$\alpha _n$$ satisfies:

$$\left\{ \begin{array}{ll} \alpha _1 \le \beta _0(1+\gamma _0) + \varrho _0, \alpha _n \le \beta _0\prod _{k=0}^{n-1}(1+\gamma _k) \\ \quad + \sum _{k=0}^{n-2} \varrho _k \prod _{r=k+1}^{n-1} (1+\gamma _r) + \varrho _{n-1}, ~~~~ n>1 \end{array}\right. $$

and, if $\beta _0\ge 0$ and $\varrho _0\ge 0$, then, we have the following inequality:

$$\alpha _n \le \Bigg (\beta _0 + \sum _{k=0}^{n-1} \varrho _k\Bigg )\exp \Bigg (\sum _{k=0}^{n-1}\gamma _k\Bigg ), ~~~ n>1.$$

### Theorem B.3

Let us assume that $\lim _{\tau \rightarrow t^{-}} \Psi [S(t), t| S(\tau ),$ $\tau ] = 0$, and denote the difference between the actual solution and the approximate solution by:

$$\begin{aligned} \varepsilon _{i}&= g[S(i\Delta t), i\Delta t|y_0, 0] - \tilde{g}[S(i\Delta t), i\Delta t|y_0, 0], \nonumber \\&\qquad i = 1, 2, ~\dots ~, K. \end{aligned}$$

Then, we have:

$$\lim _{\Delta t \rightarrow 0^{+}} |\varepsilon _{i}| = 0, ~~~~~~~~~i = 1, 2, ~\dots ~, K.$$

### Proof

The exact solution of the integral equation (10) at time step $t_i = i\Delta t$ for $i = 1, 2, ~\dots ~, K,$ is as follows:

29 $$\begin{aligned} g[S(i\Delta t), i\Delta t|y_0, 0] =&- 2 \Psi [S(i\Delta t), i\Delta t, |y_0, 0] \\&+ 2\int _0^{i\Delta t} g[S(\tau ), \tau |y_0, 0] \nonumber \\&\quad \Psi [S(i\Delta t), i\Delta t | S(\tau ), \tau ] d\tau . \nonumber \end{aligned}$$

So, by comparing the actual solution (29) and the approximate solution (16) at the first time step we have:

$$\varepsilon _{1} = 2 \int _{0}^{\Delta t} g[S(\tau ), \tau |y_0, 0] \Psi [S(\Delta t), \Delta t | S(\tau ), \tau ] d\tau .$$

Besides, for the next time steps by comparing the actual solution (29) and approximate solution (17), the error terms are obtained as follows:

$$\begin{aligned} \varepsilon _{i}&= 2 \int _{0}^{i \Delta t} g[S(\tau ), \tau |y_0, 0] \Psi [S(i\Delta t), i\Delta t | S(\tau ), \tau ] d\tau \nonumber \\&\quad - 2 \Delta t \sum _{j=1}^{i-1} \tilde{g}[S(j\Delta t), j \Delta | y_0, 0 ] \Psi [S(i\Delta t), i \Delta t | S(j\Delta t), j \Delta t]\nonumber \\&\quad i = 2, 3, ~\dots ~, K. \nonumber \end{aligned}$$

By denoting the $\delta _{1} = \varepsilon _{1}$ and $\delta _{i}$ (for $i = 2, 3, ~\dots ~, K$) as below:

$$\begin{aligned} \delta _{i}&= 2 \int _{0}^{i \Delta t} g[S(\tau ), \tau |y_0, 0] \Psi [S(i\Delta t), i\Delta t | S(\tau ), \tau ] d\tau \\&\quad - 2 \Delta t \sum _{j=1}^{i-1} g[S(j\Delta t), j \Delta | y_0, 0 ] \Psi [S(i\Delta t),\\&\quad i \Delta t | S(j\Delta t), j \Delta t] ~~~~~~~~~i = 2, 3, ~\dots ~, K, \end{aligned}$$

the error terms can be written as follows:

$$\begin{aligned} \varepsilon _{i}&= 2 \Delta t \sum _{j=1}^{i-1} \varepsilon _{j} \Psi [S(i\Delta t), i \Delta t | S(j\Delta t), j \Delta t] \\&\quad + \delta _{i} ~~~~~~~~~i = 2, 3, ~\dots ~, K. \end{aligned}$$

By defining $\xi _{K}$ and *L* in the following way:

$$\xi _{K} = \max _{1 \le i \le K} |\delta _{i \Delta t}|,$$

and

$$L = 2 \max _{0 \le \tau \le t \le T_{max}} |\Psi [S(t), t| S(\tau ), \tau ]|,$$

it is clear that:

$$|\varepsilon _{1}| \le \xi _{K}, ~~~~~ and ~~~~~ |\varepsilon _{i}| \le \Delta t L \sum _{j=1}^{i-1} |\varepsilon _{j}| + \xi _{K} ~~~~~~~~~i = 2, 3, ~\dots ~, K.$$

Then, by using the Grönwall’s inequality lemma B.1 we obtain:

$$|\varepsilon _{i}| \le \xi _{K} \exp (i \Delta t L), ~~~~~~~~~i = 1, 2, ~\dots ~, K.$$

Now, it is enough to show that $\lim _{\Delta t \rightarrow 0^{+}} \xi _{K} = 0$. We can write the following inequality for the $|\delta _i|$:

$$\begin{aligned} |\delta _{i}|&\le 2 i \Delta t ~\omega [(\Psi g)_{i\Delta t}, \Delta t/2] \le 2 K \Delta t ~\omega [(\Psi g)_{i\Delta t},\\&\quad \Delta t/2], ~~~~~~~~~i =1, 2, 3, ~\dots ~, K, \end{aligned}$$

in which $\omega$ is defined as $\omega (f, h) = \sup _{|x-x'| \le h} |f(x) - f(x')|$ and $(\Psi g)_{i\Delta t}$ is defined as follows:

$$(\Psi g)_{i\Delta t} = \Psi [S(i\Delta t), i\Delta t| S(\tau ), \tau ] g[S(\tau ), \tau | y_0, 0].$$

Thus, based on the definition of $\delta _i$, one can write:

$$\xi _{K} \le 2 K \Delta t ~\omega [(\Psi g)_{K\Delta t}, \Delta t/2].$$

Therefore, if $\Delta t \rightarrow 0$, then $\xi _{K} \rightarrow 0$ and the proof is complete. $\square$

## Appendix C Parameter recovery based on the Bessel series

Figure 12 illustrates the parameter recovery results based on the Bessel series method for fixed threshold HSDMs using 250 trials. The simulation setup was exactly similar to the simulation setup for the integral equation method. These results indicate that although the Bessel series may be unstable in certain parts of the parameter space, it remains stable in most parts, ensuring reliable parameter recovery.

## Appendix D Effect of time step on precision of parameter recovery

This appendix contains the plots of parameter recovery of three- and four-dimensional constant threshold HSDM for different values of $\Delta t$. Figures 13 and 14 illustrate the effect for three- and four-dimensional, respectively. Similar to the results reported in the main text for the circular diffusion model, here, for the higher values of the $\Delta t$ (particularly $\Delta t \ge 0.1$), the integral equation method overestimates the threshold and non-decision time. Bias in the estimation of the drift rate only occurs for drift elements with high absolute values. By decreasing the value of $\Delta t$, we can improve the parameter recovery. In particular, when $\Delta t = 0.02$, there is no bias in the parameter estimations.

## Appendix E Across trial variability

In the presence of across-trial variability in drift rate, the Girsanov change-of-measure formula becomes as follows (Smith & Corbett, 2019):

$$\begin{aligned} M(T)&= \prod \limits _{i=1}^{n} \frac{1}{\sqrt{(\eta /\sigma )^2 T + 1}}\\&\quad \times \exp \Big [-\frac{\mu _i^2}{2\eta ^2} + \frac{\big (X_i(T) (\eta /\sigma )^2 + \mu _i\big )^2}{2\eta ^2\big ((\eta /\sigma )^2 T + 1\big )}\Big ], \end{aligned}$$

in which, $\mu _i$ is the mean drift rate and $\eta$ is the across-trial drift rate variability. To evaluate parameter recovery in the presence of across-trial variability, we also did a parameter recovery study for the constant-threshold model with across-trial variability in drift rate. So, for each parameter set, which is randomly drawn from the following uniform distributions, 500 trials are generated, and then, the parameter recovery based on the integral equation method is done:

$$\begin{aligned} b&\sim \mathcal {U}[1, 3], ~~~~~~ t_0 \sim \mathcal {U}[0.1, 1], ~~~~~~ \mu _i \sim \mathcal {U}[-2.5, 2.5],\\&\quad ~~~~~~ \eta _i \sim \mathcal {U}[0.1, 1]. \end{aligned}$$

Figure 15 shows the parameter recovery results when there exists across-trial variability in the drift rate. Interestingly, all of the main parameters of the model are still recoverable with high precision. However, the recovery of the variability parameter $\eta$ is not reliable. The correlation between the actual $\eta$ and estimated $\eta$ parameter is relatively good. However, the variability in the estimation of this parameter is fairly high. Boehm et al. (2018) also reported the same issue with estimating across-trial variability of the drift rate in the standard DDM model and showed that for a reliable estimation of the variability parameter, thousands of trials are required.

## Appendix F Nonlinear collapsing thresholds

Some theoretical studies have demonstrated that, in certain circumstances, the nonlinear collapsing threshold represents an optimal strategy for decision-making (Frazier & Yu, 2007; Fudenberg et al., 2018). It is proposed that the threshold should exhibit a nonlinear, monotonically decreasing dynamic. There are lots of functions that can satisfy these conditions. However, hyperbolic and exponential functions are two functions that are frequently used in the literature (e.g., Fudenberg et al., 2018; Hanks et al., 2011; Voskuilen et al., 2016):

$$\begin{aligned} b_{Hyperbolic}(t)&= \frac{b_0}{1 + \lambda t}, b_{Exponential}(t) = b_0 \exp (-\lambda t). \end{aligned}$$

Figures 16 and 17 illustrate the compatibility of the marginal distribution of response time approximated by the integral equation method and the simulated data. The simulated data and the approximated likelihood are compatible in both cases.

## Appendix G Fokker–Planck equation for HSDM

It is well known that the transition probability density function of the stochastic process (2) satisfies the following forward Fokker–Planck equation (also known as forward Kolmogorov equation; Øksendal, 2013):

30 $$\begin{aligned} \frac{\partial }{\partial t} p(\textbf{x}, t)= &  -\sum _{i=1}^{n} \mu _i \frac{\partial }{\partial x_i} p(\textbf{x}, t) + \frac{\sigma ^2}{2}\sum _{i=1}^{n}\frac{\partial ^2}{\partial x_i^2} p(\textbf{x}, t),\nonumber \\ &  \textbf{x} \in \Omega = \{\textbf{x} \in \mathbb {R}^n: \Vert \textbf{x}\Vert <b(t)\}, \end{aligned}$$

subject to the following initial and boundary conditions:

31 $$\begin{aligned} p(\textbf{x}, 0) = \delta (\textbf{x}), \end{aligned}$$

32 $$\begin{aligned} p(\textbf{x}, t) = 0, ~~~~~ \textbf{x} \in \partial \Omega , \end{aligned}$$

where $\delta (.)$ represents the Dirac delta function. To obtain the first-passage time distribution, one can first approximate the solution of (30) and then obtain the survival probability function, $\zeta (t)$, by integrating over all spatial dimensions:

33 $$\begin{aligned} \zeta (t) = \int _{\Omega } p(\textbf{x}, t) d\textbf{x}. \end{aligned}$$

The survival probability function shows the probability that the accumulator did not reach the decision threshold up to time *t*. By taking the temporal derivative of the survival probability function, the first-passage time distribution is obtained as follows:

34 $$\begin{aligned} g(t) = -\frac{d}{dt} \zeta (t). \end{aligned}$$

Since the equation (30) is defined in an *n*-dimensional space (i.e., $\textbf{x} \in \mathbb {R}^n$), approximating the solution of this equation can be challenging. Consequently, we need more complex numerical methods such as meshless methods (Kazem et al., 2012) or splitting method (Moayeri et al., 2022). While these numerical approximation methods can be very precise in estimating the solution of the equation (30), they are computationally expensive and very time-consuming compared to the proposed integral equation method.

## Footnotes

1 Here $'$ means the transpose of the vector.

2 HSDM assumes that *n* independent components are involved in the accumulation process, which means there is no correlation between the components. A more general model can relax this assumption and consider a correlating *n*-dimensional process. However, the correlating process becomes more complex and less mathematically tractable.

3 In a more general case, HSDM can also have starting point bias (Smith, 2016), which implies that the accumulation process can be started from any interior point of the hyper-sphere.

4 See Appendix A for an intuitive explanation. Also, we refer interested readers to chapter 8 in Øksendal (2013) for a detailed discussion on the Girsanov change-of-measure theorem.

5 Smith (2023) has utilized the Feller-type process for explaining the underlying constructs of the decision process.

6 In practice, the longest response time can be considers as $T_{max}$. Also for $y_0$ we can consider a very small value like $10^{-4}$ or $10^{-6}$.

7 A tutorial on how to use the integral equation method for estimating parameters of HSDM is provided on https://github.com/AmirHoseinHadian/IE4HSDM.

8 The same parameter recovery results are also presented for the Bessel series method in Appendix C. Although the Bessel series is unstable for some parameter values, it has a good parameter recovery performance. However, the parameter recovery performance of the integral equation method is slightly better.

9 This longer computational time is caused by computing fractional-order Bessel functions (i.e., $\nu =\frac{1}{2}$) and their roots. In other words, $\nu = \frac{1}{2}$ in series (6) for three-dimensional HSDM. However, for two-dimensional and four-dimensional HSDMs, the Bessel functions are integer order (i.e., $\nu =0$ for 2D and $\nu =1$ for 4D).

10 See Appendix A for an intuitive example of how this theorem can be utilized to obtain the first-passage time distribution of the single collapsing threshold Wiener process.

11 Note that there are some attempts to make the PDA approach more efficient by using parallelization (e.g., Lin et al., 2019).

12 For a discussion of the benefits of the Bayesian parameter estimation methods, see Lee (2011).
